# A Homogeneously Catalyzed Paired Electrolytic Cell for Hydrogen Peroxide Production

**DOI:** 10.1002/anie.202524811

**Published:** 2026-03-23

**Authors:** Caterina Trotta, Jesse Orta, Hendrik C. de Heer, Pim G. J. van Duren, Maxime A. Siegler, Gabriel Menendez Rodriguez, Alceo Macchioni, D. G. H. Hetterscheid

**Affiliations:** ^1^ Department of Chemistry Biology and Biotechnology and CIRCC University of Perugia Perugia Italy; ^2^ Leiden Institute of Chemistry Leiden University Leiden The Netherlands; ^3^ Department of Chemistry Johns Hopkins University Baltimore Maryland USA

**Keywords:** hydrogen peroxide, paired electrolysis, water oxidation, oxygen reduction, sn porphyrins

## Abstract

The two‐electron water oxidation reaction (2e^−^‐WOR) and oxygen reduction reaction (2e^−^‐ORR) represent sustainable and promising processes for the electrochemical synthesis of hydrogen peroxide (H_2_O_2_). The main factor hampering the realization of a paired electrochemical cell for H_2_O_2_ production is finding appropriate catalysts for both 2e^−^‐ORR and 2e^−^‐WOR, able to work under the same experimental conditions. Herein we show that **Cu(tmpa))** and **Sn‐TMPyP** are compatible and efficient catalysts for 2e^−^‐ORR and 2e^−^‐WOR, respectively. They have been used to assemble a paired electrochemical cell for H_2_O_2_ production. The latter exhibits a total overpotential of 570 mV, distributed between the two electrodes. During a 3 h bulk electrolysis experiment, the cathodic Faradaic efficiency ranged from 15% to 19% with a H_2_O_2_ production rate of 1.6 µmol h^−^^1^ cm^−^^2^. Meanwhile, at the anode, the Faradaic efficiency stabilized between 40% and 50%, yielding a H_2_O_2_ production rate of 3.5 µmol h^−^^1^ cm^−^^2^. The remarkable activity of **Sn‐TMPyP** as a catalyst for the 2e^−^‐WOR, ranking among the highest reported for molecular catalysts, is ascribed to the selection of a carbonate buffer as the electrolyte, which enhanced catalytic performance by facilitating dissociation of H_2_O_2_ from the Sn catalyst. This work establishes a new benchmark for homogeneous dual‐electrode H_2_O_2_ electrosynthesis.

## Graphical abstract

**Cu(tmpa)** and **Sn‐TMPyP** are compatible and efficient catalysts for 2e^−^‐ORR and 2e^−^‐WOR, respectively, which have been employed to assemble an efficient paired electrochemical cell for H_2_O_2_ production. The presence of a carbonate buffer is essential to achieve a high activity in the anode compartment, while control over mass transport is essential for a good Faradaic efficiency in the cathode compartment.

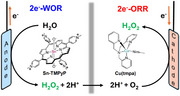

## 1 Introduction

Hydrogen peroxide is a valuable bulk chemical, extensively employed as a green oxidant given its ability to oxidize a variety of substrates releasing water as the only byproduct [1]. It is also gaining significant attention as energy carrier, since it can be used as catholyte in specific types of fuel cells [2, 3]. Currently, the majority of hydrogen peroxide is industrially produced in organic solvents *via* the anthraquinone process, a batch process that necessitates multiple purification and concentration steps to yield highly concentrated hydrogen peroxide solutions [4]. However, the transportation and handling of such concentrated solutions poses significant safety challenges. Furthermore, most of the applications necessitate the use of diluted solutions.

The in situ and on‐demand electrochemical synthesis of H_2_O_2_ from aqueous solutions is an extremely appealing and sustainable alternative [5, 6]. It can be achieved through two diverse pathways, namely the 2e‐oxygen reduction reaction (2e^−^‐ORR, Equation 1) and the 2e‐water oxidation reaction (2e^−^‐WOR, Equation 2):

(1) $$O_{2}+2H^{+}+2e^{-}\to H_{2}O_{2}E=0.67\;V\;\mathrm{vs}.\;\mathrm{RHE}$$

(2) $$2H_{2}O\to H_{2}O_{2}+2H^{+}+2e^{-}E=1.76\;V\;\mathrm{vs}.\;\mathrm{RHE}$$

The primary challenge in the electrochemical synthesis of H_2_O_2_ is related to selectivity [7, 8, 9]. The standard potential for the formation of H_2_O_2_ through reaction 2 is more positive than that required for the further oxidation of H_2_O_2_ to O_2_. Consequently, the produced H_2_O_2_ may be easily lost due to overoxidation on the electrode surface. Thermodynamics also plays a critical role in the selective cathodic electrosynthesis of H_2_O_2_ because the reduction of O_2_ to H_2_O_2_ has a more positive standard potential than that of the hydrogen peroxide reduction reaction (HPRR) and of the 4 electrons reduction of O_2_ to H_2_O. Hence, these three processes compete. It follows that the choice of appropriate catalytic systems to selectively target ORR and WOR to H_2_O_2_ is of fundamental importance.

Homogeneous catalysts are highly tuneable, allowing their electronic and steric properties to be precisely adjusted and thereby enabling very low overpotentials. Many complexes based on first‐row transition metals, including Co [10, 11, 12, 13, 14, 15] porphyrins, Fe [16, 17, 18, 19, 20] and Cu [21, 22, 23, 24] complexes have been proposed as fast and selective catalysts for the 2e^−^‐ORR. Particularly cobalt porphyrin and cobalt salen complexes are well known to produce H_2_O_2_ with good Faradaic efficiencies ($FE_{H_{2}O_{2}}$). Although catalysts with remarkable performances in terms of selectivity and TOF have been reported, these studies have rarely been extended beyond a single linear sweep experiment and their durability under prolonged bulk electrolysis conditions has thus far remained unsatisfactory [25, 26]. [Cu(tmpa)(OH_2_)](OTf) [tmpa = tris(2‐pyridylmethyl)amine, **Cu(tmpa)**] is known to be an efficient catalyst for ORR that follows a stepwise 4e^−^/4H^+^ pathway, in which H_2_O_2_ is a detectable intermediate, subsequently reduced to H_2_O through HPRR [27, 28, 29, 30]. The selectivity of this catalyst for H_2_O_2_ has been rigorously evaluated through rotating ring‐disk electrode (RRDE) experiments, which revealed that it depends on two factors: (i) the ratio of the rate constants for the 2e^−^‐ORR and the HPRR, which determines whether H_2_O_2_ accumulates or is further reduced to water and (ii) the relative concentrations of O_2_ and H_2_O_2_ near the electrode surface, which affects the dynamics of the reaction. As the local H_2_O_2_ concentration increases, the probability of its further reduction also increases, resulting in a lower selectivity. Detailed experiments showed that the maximum selectivity for H_2_O_2_ ($FE_{H_{2}O_{2}}$ of 80%) is obtained at low catalyst concentrations and near the onset potential, where HPRR is negligible compared to ORR and H_2_O_2_ accumulation is restrained. Additionally, controlled potential electrolysis (CPE) experiments in phosphate buffer solution (PBS) at pH 7 confirmed that this catalyst can stably generate H_2_O_2_ with $FE_{H_{2}O_{2}}$ of 50% for 8 h, without loss of activity and that its efficiency is only marginally affected by the composition of the electrolyte employed, as long as the pH is not too strongly acidic or alkaline [30].

Compared to the 2e^−^‐ORR, homogeneous 2e^−^‐WOR has been much less investigated [31, 32]. Indeed, only a few molecular metalloporphyrins bearing TMPyP [5,10,15,20‐tetrakis(1‐methyl‐4‐pyridinio)porphyrin] have been reported for the anodic electrosynthesis of H_2_O_2_ [33, 34, 35, 36]. Yet, in terms of overpotential, molecular catalysts for 2e^−^‐WOR have shown superior performances over heterogeneous catalysts, which often require overpotentials exceeding 1 V [37]. Among these molecular metalloporphyrins, **Sn‐TMPyP** catalyses the 2e^−^‐WOR under the widest range of pH, with an overpotential of 370 mV and $\;FE_{H_{2}O_{2}}$ of 18% over 3 h bulk electrolysis experiment in Na_2_SO_4_ at pH 8.5 [34]. The protolytic behavior of the axial water molecules of **Sn‐TMPyP** is well documented and the catalytically active species, bearing doubly deprotonated axial oxide ligands, is produced at pH above 4.3, allowing for its versatile application in neutral or slightly basic electrolytes (see Supporting Information) [38]. Despite the catalytic versatility, this complex has received limited attention with key factors like electrolyte composition, potentially crucial for further optimization [39], remaining largely unexplored.

Once the appropriate catalysts have been identified, a step forward can be made in the realization of an electrocatalytic system for H_2_O_2_ production: by pairing ORR with WOR in a single apparatus, which can be fed by electricity coming from renewable sources, productivity can be maximized owing to the concurrent production of H_2_O_2_ at both electrodes. Whereas a few of such paired electrocatalytic systems based on the heterogeneous catalysts have been reported [40, 41, 42, 43, 44], to the best of our knowledge, systems based on homogeneous catalysts are unknown. This is likely due to the substantially different reaction conditions under which homogeneous catalysts for 2e^−^‐ORR and 2e^−^‐WOR operate, posing compatibility issues. Furthermore, as observed for **Cu(tmpa)** and a few other catalysts [30], 2e^−^‐ORR catalysts frequently encounter difficulties in sustaining adequate H_2_O_2_ production over extended periods. Implementing a paired electrocatalytic systems based on homogeneous catalysts would be extremely important, also for providing a better understanding of reaction mechanisms and evidencing the critical factors to be addressed to improve performance [45, 46, 47].

**FIGURE 1:**
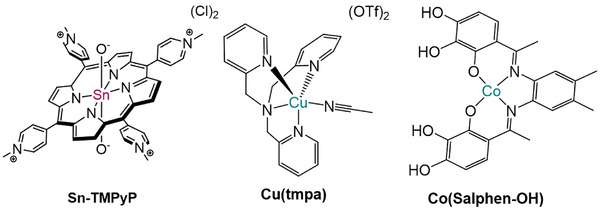
Schematic structure of **Sn‐TMPyP**, **Cu(tmpa)** and **Co(Salphen–OH)**.

In this study we developed an electrochemical cell employing molecular catalysts to produce H_2_O_2_ both at the cathode and the anode. **Cu(tmpa)** and **Co(Salphen–OH)** have been selected as catalysts for the 2e^−^‐ORR (Figure 1) [13, 30], as **Cu(tmpa)** is characterized by fast kinetics, great long‐term stability and high compatibility with various electrolytes, even if its selectivity is generally controlled by mass transport phenomena; **Co(Salphen–OH)** was shown to be the most promising Co‐catalyst in a direct comparison with **Co(Salphen–OMe), Co‐OEP**, **Co‐TMPyP, Co(Salen)** and **CoTPP** (*H_2_* Salphen–OH = *N,N′*‐Bis‐4,3‐(dihydroxysalicylidene)‐4,5‐xylenylenediamine; *H_2_* Salphen–OMe = *N,N′*‐Bis‐4,6‐(dimethoxysalicylidene)‐4,5‐xylenylenediamine; *H_2_* OEP = octaethylporpyrin; *H_2_* Salen = N,N’‐bis(salicylidene)ethylenediamine; *H_2_* TPP = tetraphenylporphyrin). On the other side, **Sn‐TMPyP** was chosen as catalyst for the 2e^−^‐WOR given its ability to be active in a wide range of pH, addressing the essential point of increasing its activity and selectivity. In this study we have pinpointed the dissociation of H_2_O_2_ from the Sn catalyst as the rate determining step, which can be significantly boosted by a tailored choice of the electrolyte composition, that opens a new pathway for the release of the product from a stable reaction intermediate. Indeed, the $FE_{H_{2}O_{2}}$ of **Sn‐TMPyP** was significantly increased to reach over 50% and a total productivity of 6 µmol h^−1^ cm^−2^ was achieved in bulk electrolysis experiments, using carbonate buffer as electrolyte. This selectivity at the anode was largely maintained in a convergent paired electrolysis setup, in which **Cu(tmpa)** or **Co(Salphen–OH)** was used to cathodically produce H_2_O_2_ from O_2_, thereby representing the first example of homogeneously catalyzed simultaneous electrosynthesis of hydrogen peroxide in an electrochemical cell.

## 2 Results and Discussion

**FIGURE 2:**
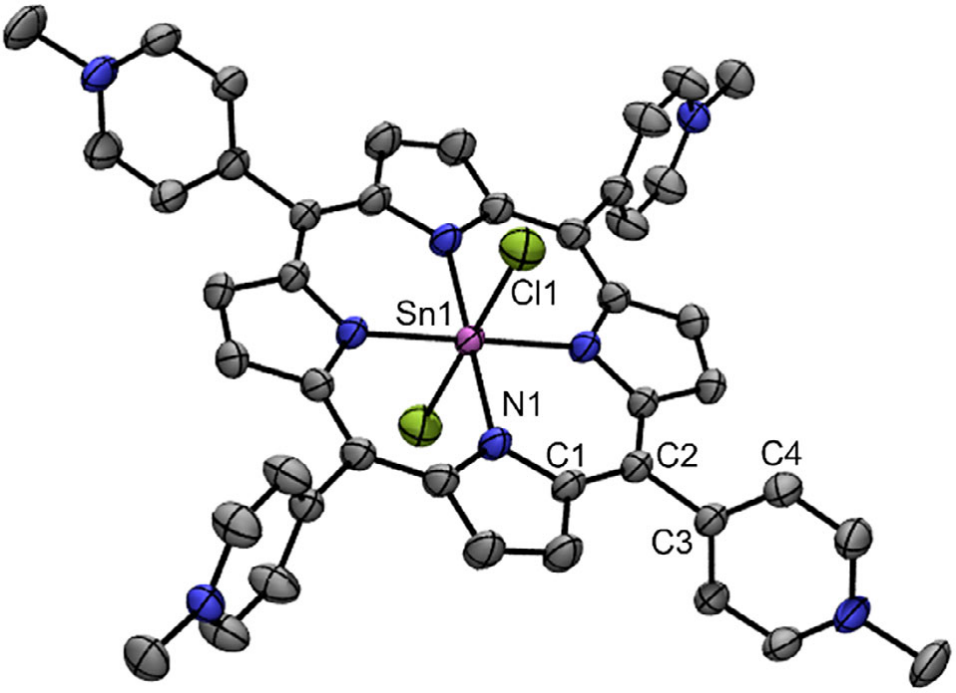
Displacement ellipsoid plot (50% probability level) of [Sn^IV^(Cl)_2_(TMPyP)]Cl_2_ at 223(2) K. Hydrogen atoms and counterion are omitted for clarity. Color code: Sn = pink, N = blue, Cl = green, C = gray. Relevant distances (Å) and angles (°): Sn1─Cl1 = 2.4235(7), Sn1─N1/N2 = 2.0858(19)/2.097(2), Cl1‐Sn1‐N1 = 89.81(4), Sn1‐N1‐C1 = 125.73(13), C1‐C2‐C3 = 116.73(18), C2‐C3‐C4 = 120.80(19).

**Sn‐TMPyP** was synthesized according to a literature procedure [48]. Its purity was confirmed by ^1^H NMR and LC‐MS analysis (see Supporting Information). Single crystals suitable for X‐ray diffraction studies were obtained by slow diffusion of diethyl ether in a concentrated solution of **Sn‐TMPyP** in methanol and the structure is shown in Figure 2 [49].

Sn^IV^ is positioned in the center of the TMPyP porphyrin ring and is coordinated to two axial chloride atoms with bond lengths of 2.42 Å, in analogy with bond lengths reported for similar Sn^IV^ containing porphyrins (2.42 Å) [50]. The Sn─N1 bond length is 2.097 Å, in line with the bond length found for the Sn^IV^ porphyrin bearing 5,15‐bispyridine‐10,20‐benzonitrylporphyrin (2.094 Å), thereby suggesting that a modification on the porphyrin‐substituent environment only slightly affects the metal core bond lengths [50]. The Sn─N1 bond is slightly longer than that found in the crystal structure of analogous Cu^II^‐TMPyP(OTf)_2_ (2.003 Å) [51], accordingly with the presence of strongly coordinating axial chloride ligands. Interestingly, the *meso* substituents are tilted with respect to the porphyrin plane with dihedral angles of 73.74° for **Sn‐TMPyP** which are similar to those of Cu^II^‐TMPyP(OTf)_2_ (79.03°), meaning that the orientation of the substituents is mildly affected by the nature of the central metal [51].

**FIGURE 3:**
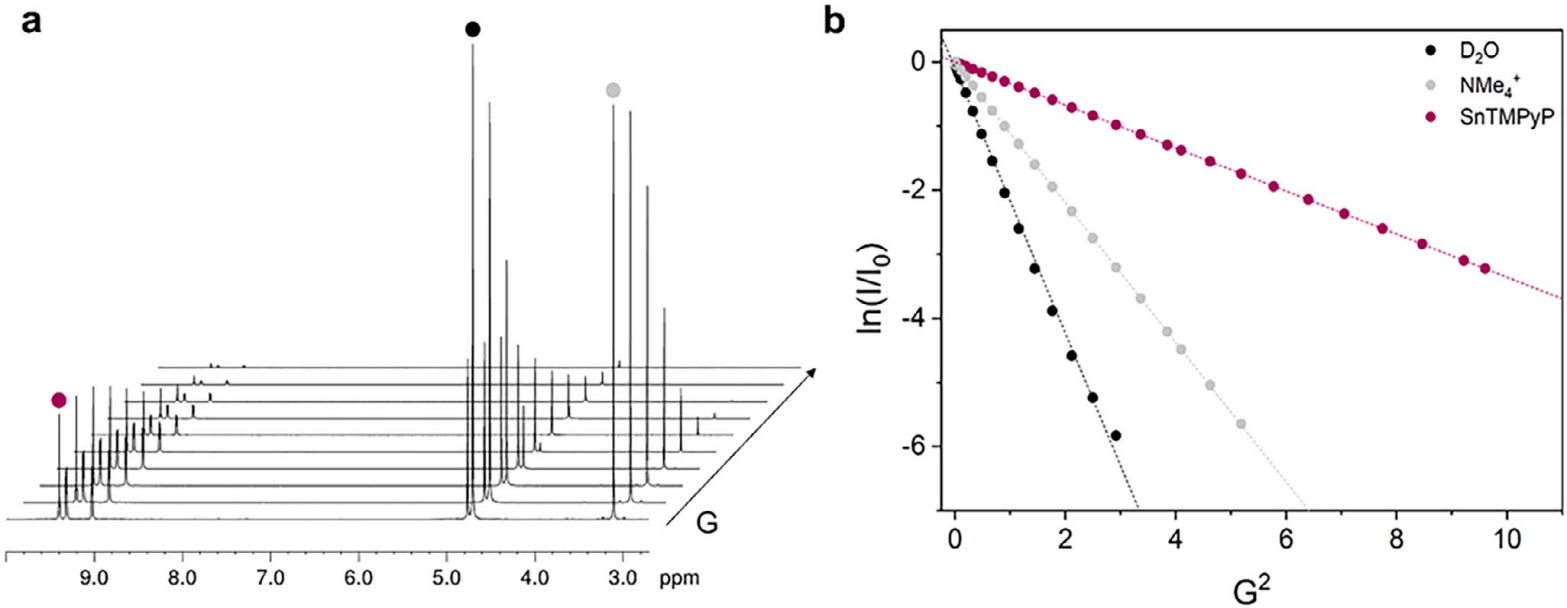
a) ^1^H NMR spectrum as a function of the pulse field gradient G and b) semi logarithmic plot of I/I_0_ versus G^2^ for a solution of **Sn‐TMPyP** and NMe_4_BF_4_ in 0.1 M deuterated carbonate buffer at pH 9.2.

Pulse Gradient Spin Echo (PGSE) NMR experiments in D_2_O with deuterated carbonate buffer were performed to assess the level of aggregation of **Sn‐TMPyP** in solution. A series of ^1^H NMR spectra were recorded by varying the strength of the pulsed field gradient (G) along the z‐axis (Figure 3a). The plot of ln(I/I_0_) (where I and I_0_ represents the intensity of resonance in the presence and absence of the pulsed field gradient, respectively) against G^2^ gives a linear correlation, from which a translational self‐diffusion coefficient (*D*_t_) of 3.08 × 10^−6^ cm^2^ s^−1^ was estimated (Figure 3b). Using a modified version of the Stokes–Einstein equation [52, 53, 54, 55, 56], the hydrodynamic volume of **Sn‐TMPyP** was derived (*V*_H_ = 1412 ± 212 Å^3^), which is smaller than the crystallographic volume (*V*_Xray_ = 1635 Å^3^) and about 1.7–1.8 times higher than the Van der Waals volume (*V*_VdW_ = 767 and 805 Å^3^, for the cation and entire salt, respectively), both deducible from the X‐ray structures of **Sn‐TMPyP**. These results are consistent with the predominance of the complex in solution as mononuclear species [52].

The cyclic voltammogram (CV) of **Sn‐TMPyP** (0.2 mM), recorded with a boron doped diamond (BDD) electrode in 0.1 M carbonate buffer at pH 9.2 showed the presence of two reversible redox couples located at 0.2 V and 0 V versus RHE (Figure S4). These were attributed to the 1e‐ reduction of the porphyrin ring to give a π‐anion radical and its subsequent 1e‐ reduction to afford the doubly reduced phlorine species, respectively [57, 58, 59]. The presence of similar redox events in the cyclic voltammogram of the tosylate salt of H_2_TMPyP corroborates this assignment (Figure S4). The plot of the cathodic current of the first redox couple against the square root of scan rate (Figure S5), revealed a linear correlation in accordance with the diffusive nature of this redox event. Using the Randles–Ševčik equation [60], the diffusion coefficient of **Sn‐TMPyP** was determined to be 3.10 × 10^−6^ cm^2^ s^−1^, which is in excellent agreement with that obtained by PGSE NMR spectroscopy.

**FIGURE 4:**
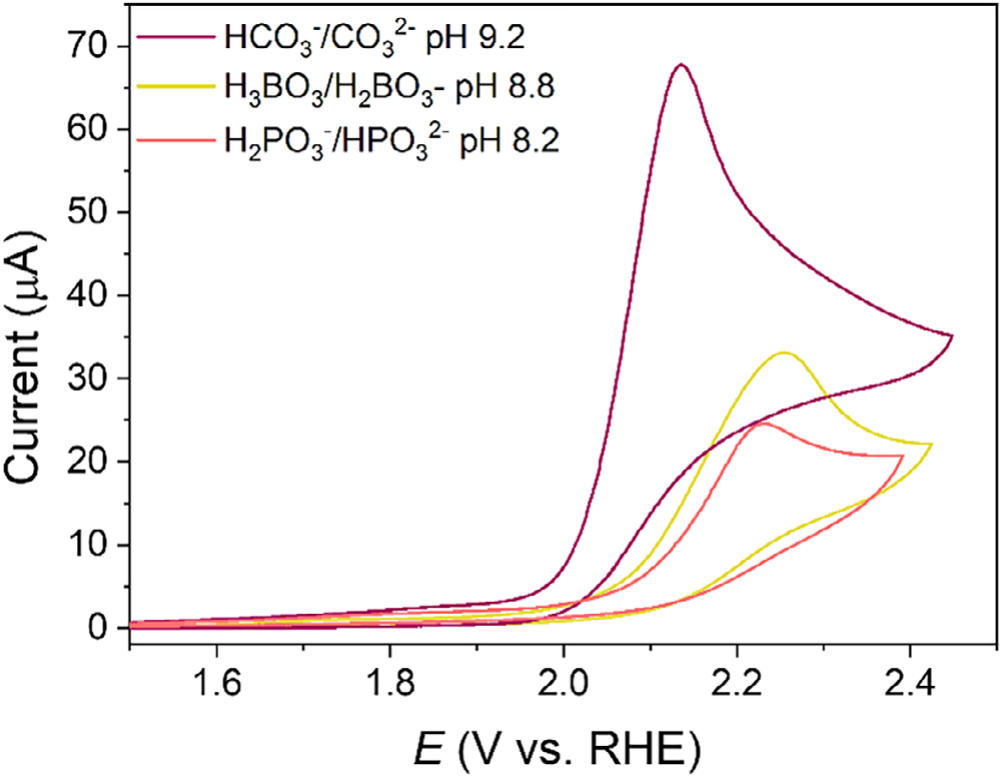
CVs of 0.2 mM **Sn‐TMPyP** in 0.1 M PBS at pH 8.2, 0.1 M borate buffer at pH 8.8 and 0.1 M carbonate buffer at pH 9.2 at 100 mV s^−1^ under Ar.

At a higher anodic potential (2.1 V versus RHE), an additional irreversible wave was observed, marked by a substantial increase in current (Figure 4). The latter process was ascribed to the 2e^−^‐WOR promoted by **Sn‐TMPyP** [34]. In order to assess whether **Sn‐TMPyP** is able to further oxidize H_2_O_2_ in the investigated potential range, CVs were recorded with increasing concentrations of hydrogen peroxide (from 0.2 to 5 mM, Figure S7). A small oxidative peak, of which the current was found to be proportional to the H_2_O_2_ concentration, emerged at 1.75 V vs. RHE and was ascribed to the oxidation of H_2_O_2_ promoted by **Sn‐TMPyP**. Nevertheless, the catalytic current at 2.1 V versus RHE was not substantially affected by the addition of H_2_O_2_ suggesting that, although the applied potential is sufficiently high to oxidize H_2_O_2_ to H_2_O, most of the catalytic current at 2.1 V can be ascribed to the 2e^−^‐WOR.

Further electrochemical tests were conducted to evaluate the effect of the composition of the supporting electrolyte. As shown in Figure 4, substantially lower catalytic currents were observed when PBS (0.1 M, pH 8.2) or borate buffer (0.1 M, pH 8.8) were utilized in place of carbonate buffer (0.1 M, pH 9.2), thereby suggesting that the presence of carbonate aids the catalysis, possibly by establishing novel pathways for the rate‐determining step of the reaction [61]. Specifically, the maximum catalytic current observed in phosphate or borate buffers reaches 25 and 32 µA, respectively, which are about half of that reached in carbonate buffer (69 µA). In addition, higher catalytic onset potentials are observed in both PBS (2.07 V versus RHE) and borate buffer (2.07 V versus RHE) than in carbonate buffer (2.00 V versus RHE). The latter corresponds to an overpotential of 240 mV for the 2e^−^‐WOR (Figure 4). The superior catalytic activity demonstrated by **Sn‐TMPyP** in carbonate buffer might be attributed to the key role of HCO_3_^−^ anion in promoting the dissociation of HOO^−^ from the metal center (*vide infra*). Similar beneficial effects of carbonate have been previously reported in the literature [40, 62, 63, 64, 65].

Before conducting further mechanistic studies we investigated whether, under catalytic conditions, **Sn‐TMPyP** is deposited on the boron doped diamond (BDD) electrode surface by recording CVs in a fresh electrolyte solution after 25 CVs in a 0.2 mM catalyst solution (Figure S6). The first CV recorded after the electrode was rinsed, revealed the presence of a small oxidative peak at 2.1 V versus RHE, with a current comparable to that of the blank of the electrode, which disappears after the second scan. Therefore, we could assume that the possible species deposited during catalysis with **Sn‐TMPyP** are not catalytically active, or that the porphyrin may be only physically adsorbed on the electrode surface.

**FIGURE 5:**
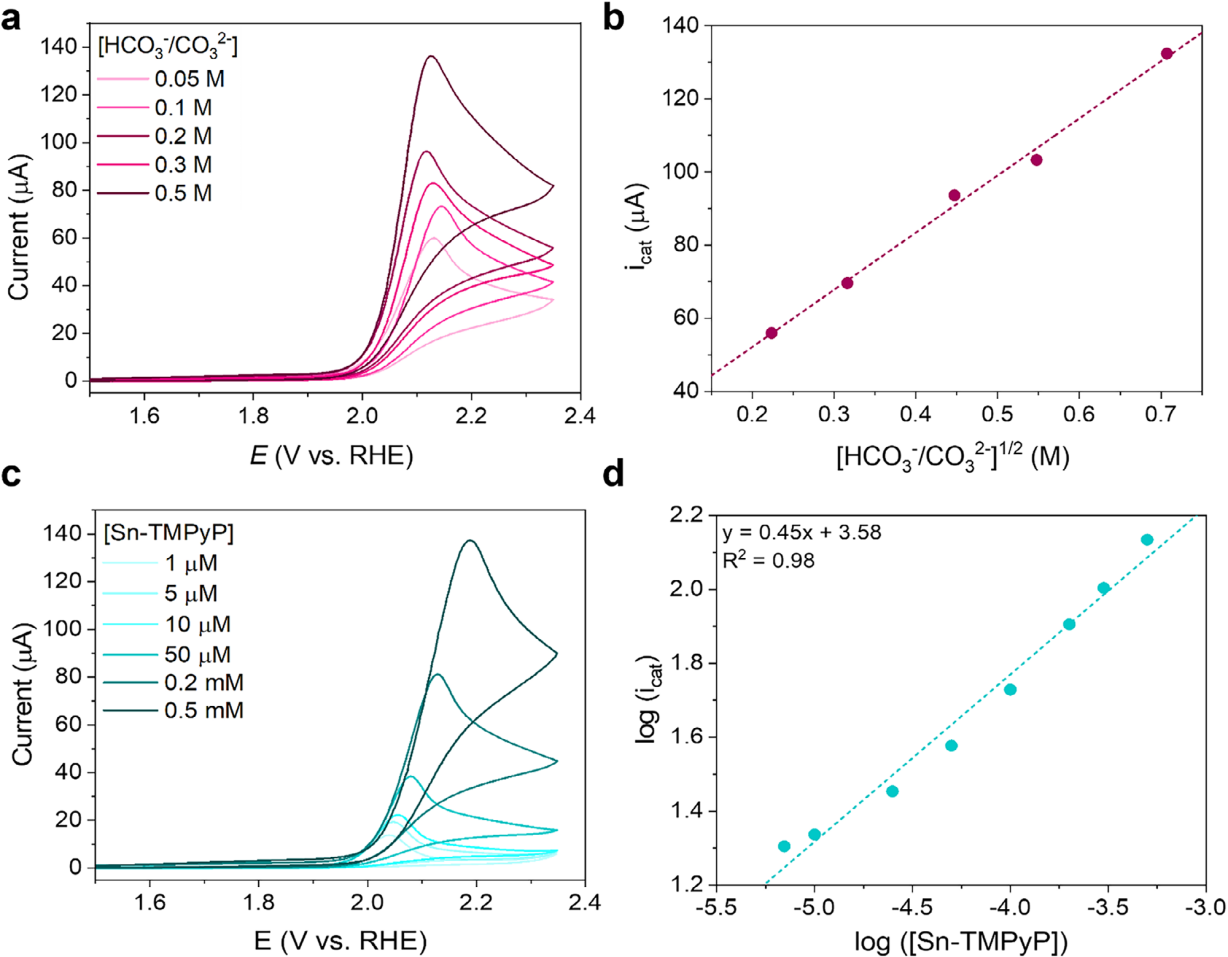
(a) CVs of 0.2 mM **Sn‐TMPyP** in 0.05–0.5 M carbonate buffer at pH 9.2 at 100 mVs^−1^ under Ar; (b) plot of the catalytic current as a function of the square root of the buffer concentration; (c) CVs of 1 µM – 0.5 mM **Sn‐TMPyP** in 0.1 M carbonate buffer at pH 9.2 at 100 mVs^−1^ under Ar; (d) logarithmic plot of the catalytic current as a function of the catalyst concentration.

To elucidate the reaction mechanism for the catalytic water oxidation, the reaction orders in buffer concentration and catalyst concentration were determined. Figure 5a shows CVs recorded in carbonate buffer at pH 9.2 in the 0.05–0.5 M concentration range, from which it is evident that the catalytic current increases with buffer concentration. To obtain the reaction order in the electrolyte concentration, the relationship between the carbonate concentration and the observed first‐order rate constant (*k*_obs_) has been examined. For a catalytic process and under pure kinetic conditions, the limit catalytic current (*i*_cat_) corresponds to Equation 3: [66]

(3) $$i_{\mathrm{cat}}=n_{\mathrm{cat}}\mathrm{FA}{\left[\mathrm{Sn}-\mathrm{TMPyP}\right]}_{0}{\left(D_{t}k_{\mathrm{obs}}\right)}^{1/2\;\;\;\;\;\;\;\;\;\;}$$

where *n*_cat_ corresponds to the number of electrons transferred in the catalytic process, *F* is the Faraday constant, A is the surface area of the electrode and *D*_t_ is the translational diffusion constant for the catalyst in this electrolyte. The presence of a linear correlation between the observed catalytic current and the square root of the carbonate buffer concentration confirms that the electrolyte contributes to the rate constant (*k*_obs_) and is engaged in the rate determining step of the reaction (Figure 5b). Similarly, the reaction order in catalyst concentration was determined by recording CVs at **Sn‐TMPyP** concentrations between 1 µM and 0.5 mM in 0.1 M carbonate buffer at pH 9.2 (Figure 5c). A progressive increase of the catalytic current with the catalyst concentration was found. Moreover, the linear trend between *i*_cat_ and the catalyst concentration indicates that the maximum catalytic current is not affected by diffusion limitations. The order in catalyst concentration was determined by the corresponding double logarithmic plot (Figure 5d) which has a slope of 0.45, suggesting the possible presence of associative side phenomena. In light of the NMR investigation performed earlier, we could rule out that an eventual dimerization of the catalyst occurs prior to the catalytic process. DFT calculations performed by the Inoue group suggest that the Sn hydroperoxide species may undergo side reactions producing dimeric species [34]. However, we have been unable to detect such species.

**FIGURE 6:**
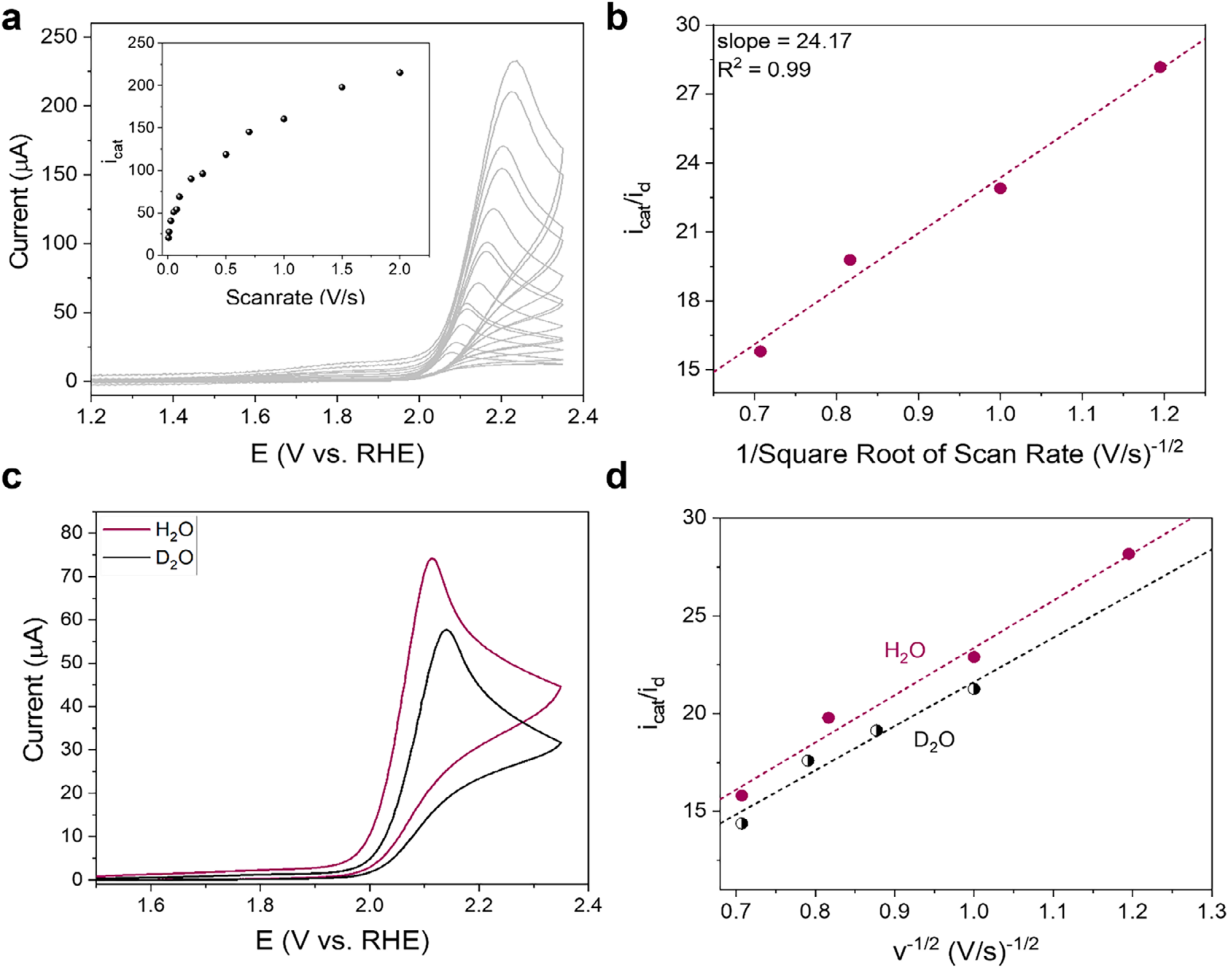
(a) CVs of 0.2 mM **Sn‐TMPyP** in 0.1 M carbonate buffer pH 9.2 at various scan rates from 5 to 2000 mVs^−1^ under Ar. The inset shows the plot of the catalytic peak current (*i*_cat_) as function of the scan rate. (b) Plot of the blank corrected i_cat_/i_d_ versus the reciprocal of the square root of scan rate; (c) CVs of **Sn‐TMPyP** (0.2 mM) in 0.1 M carbonate buffer in D_2_O and in H_2_O at pH 9.2 at 100 mVs^−1^ under Ar. (d) Plot of *i*_cat_/*i*_d_ versus the reciprocal of the square root of scan rate in D_2_O and in H_2_O.

The rate constant for the 2e^−^‐WOR to H_2_O_2_ was calculated from the plot of the catalytic current enhancement (*i*_cat_/*i*_d_) as a function of the scan rate, according to Equation 4: [39, 67].

(4) $$\;\frac{i_{cat}}{i_{d}}=\frac{n_{cat}}{0.4463}\;\sqrt{\frac{RT}{F\nu }k_{obs}}$$

where i_cat_ corresponds to the peak catalytic current and i_d_ corresponds to the cathodic peak current for the porphyrin reduction at 0 V versus RHE, R,T and F are tabulated constants, n_cat_ corresponds to the number of electrons transferred during the catalytic reaction and *ν* is the scan rate in V/s. A *k*_obs_ of 1.1 × 10^3^ ± 0.2 × 10^3^ s^−1^ was calculated through this equation (Figure 6b).

**FIGURE 7:**
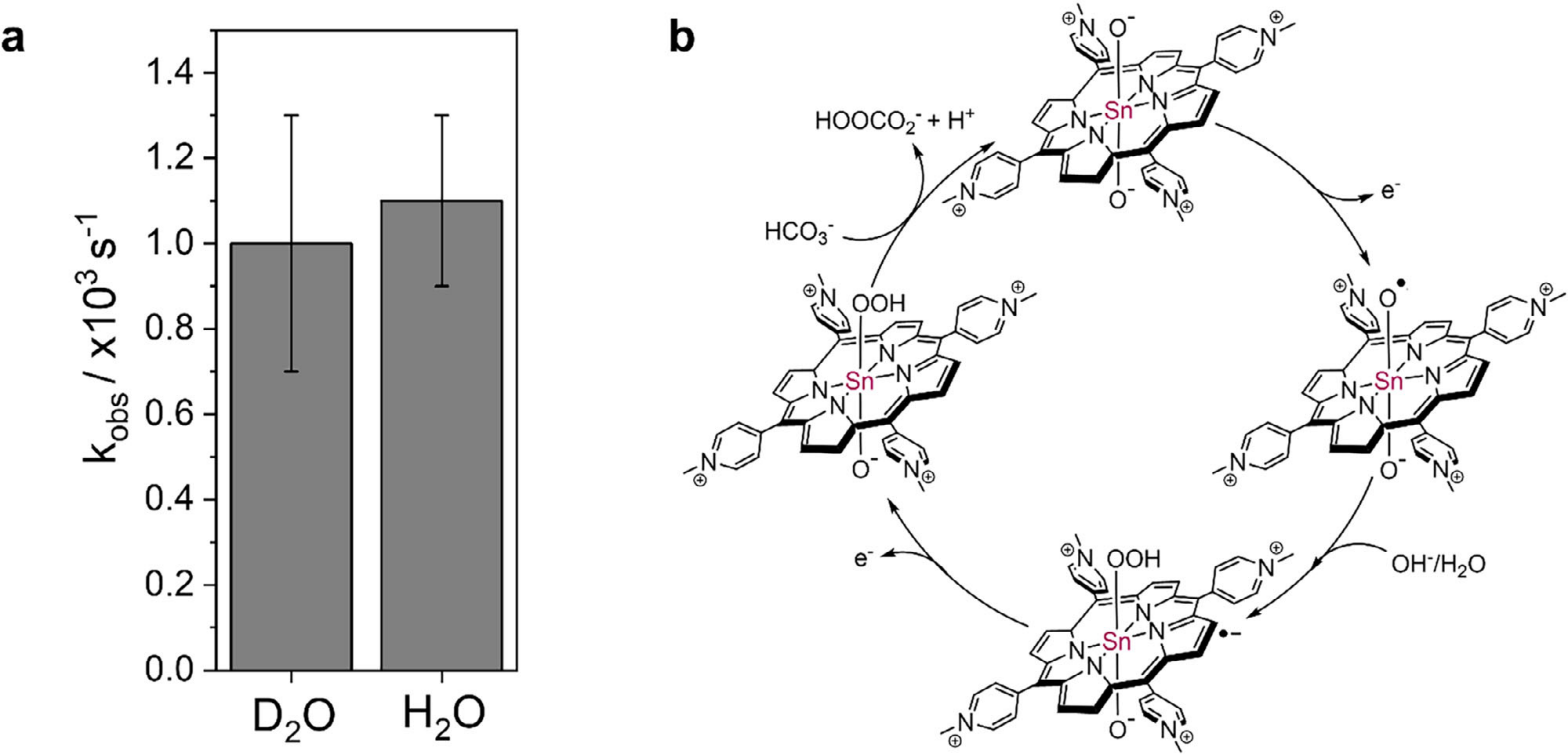
(a) Comparison of the *k*_obs_ for water oxidation in H_2_O and in D_2_O. (b) Proposed catalytic mechanism for 2e^−^‐WOR promoted by **Sn‐TMPyP**.

To gain further insights into the rate‐determining step of the mechanism of the electrocatalytic water oxidation, a solvent kinetic isotope effect (KIE) was determined by recording CVs in 0.2 mM solutions of **Sn‐TMPyP** in both deuterated and non‐deuterated 0.1 M carbonate buffer under Ar (Figure 6c). In D_2_O, the onset potential for catalytic water oxidation increased by 10 mV, reaching 2.01 V versus RHE, while the peak current decreased from 69 to 57 µA in deuterated solution. As a result, a lower k_obs_ value was obtained in D_2_O (1.0 × 10^3^ ± 0.3 × 10^3^ s^−1^), corresponding to a KIE of 1.10 (Figure 7a). For many catalytic systems, formation of the O─O bond through a base assisted water nucleophilic attack (WNA) mechanism is rate limiting [68]. Yet for such a pathway a KIE > 2 is expected [69]. The low KIE value obtained here indicates that the rate determining step of the reaction does not involve relocation of a proton. Notably, a significant enhancement of catalytic activity is observed in the presence of a carbonate buffer, with a shift in onset potential and an increase in activity significantly larger than expected from a purely Nernstian shift (Figure 4). This observation suggests that carbonate plays an active role in the rate determining step of peroxide formation. However, the absence of a measurable kinetic isotope effect implies that the effect of carbonate is not associated with proton shuttling, but rather with a different mechanistic contribution. In case of **Sn‐TMPyP**, it is expected that dissociation of peroxide is the rate limiting step of the reaction. The absence of a significant KIE could be explained by a displacement reaction involving the Sn─OOH intermediate species and either a hydroxyl or a carbonate/bicarbonate ion. Considering the experimental conditions applied (pH 9.2) and the composition of the electrolyte, the bicarbonate concentration (0.085 M) prevails over the OH^–^ concentration (1.6 × 10^−4 ^M). Moreover, the evident dependence of the catalytic current on the carbonate buffer concentration suggests that H_2_O_2_ is likely converted to hydroperoxocarbonate, owing to the higher concentration of bicarbonate over carbonate at this pH. In light of the kinetic results obtained, a reaction mechanism for 2e^−^‐WOR catalyzed by **Sn‐TMPyP** is proposed (Figure 7b).

The proposed catalytic cycle follows an ECEC mechanism. It starts with the one electron oxidation of the starting species [Sn‐TMPyP(O^−^)_2_]^2+^ to form an oxyl radical species [Sn‐TMPyP(O^−^)(O•)]^3+^. Then the attack of a hydroxide anion or of a water molecule would lead to the formation of a radical hydroperoxo species [Sn‐TMPyP•(O^−^)(OOH)]^2+^, which is further oxidized to produce [Sn‐TMPyP(O^−^)(OOH)]^3+^. In the final, rate‐determining step of the reaction, the bicarbonate ion promotes the dissociation of hydrogen peroxide from [Sn‐TMPyP(O^−^)(OOH)]^3^^+^ as hydroperoxocarbonate, leading to regeneration of the double oxo starting complex [70]. Percarbonates are readily formed in mixtures of carbonate and hydrogen peroxide [71]. We exclude a mechanism wherein carbonate radicals play a role, even though such species are thermodynamically accessible at these potentials, as we do not see any appreciable currents in carbonate buffers in absence of **Sn‐TMPyP**. Typically, the presence of hydroxyl radicals [64, 65] and much higher oxidation potentials [40] are necessary to generate such carbonate radical species.

**FIGURE 8:**
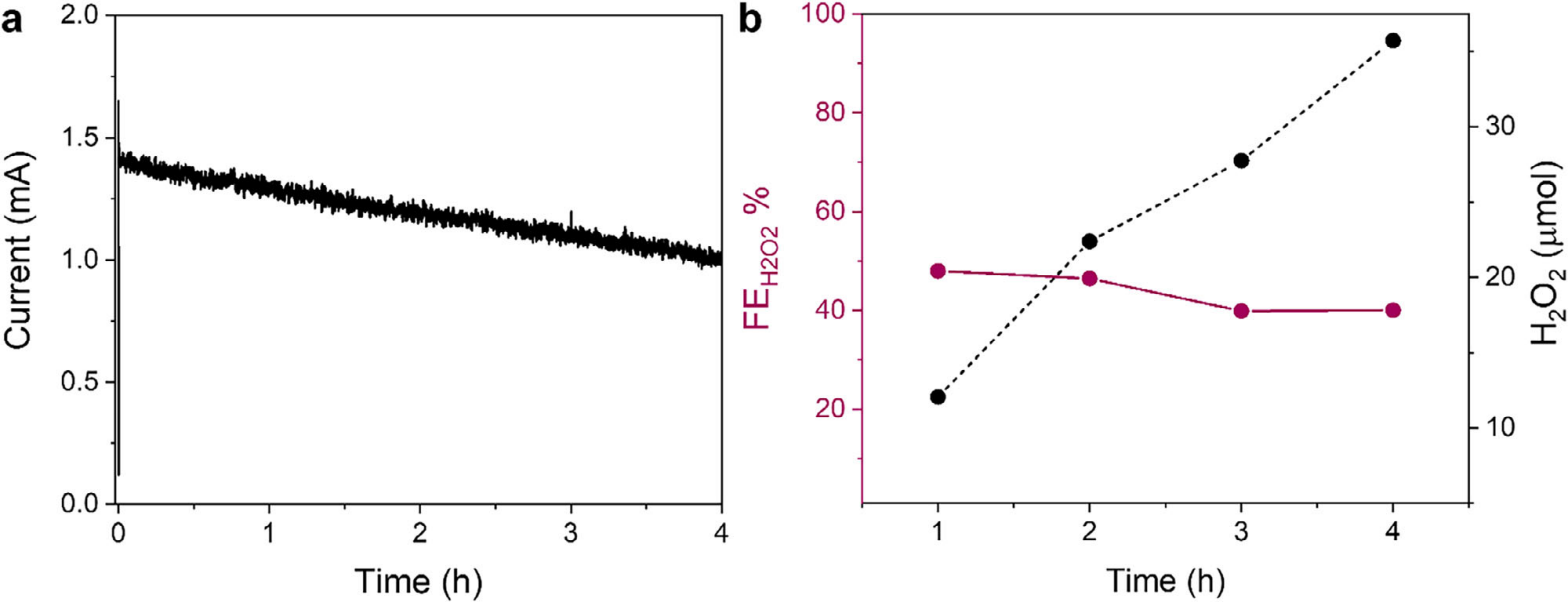
(a) Amperogram (b) $FE_{H_{2}O_{2}}$ and µmol of H_2_O_2_ produced during 4 h CPE at 2.1 V versus RHE with 0.2 mM **Sn‐TMPyP** in 0.1 M carbonate buffer at pH 9.2 under Ar.

The amount of H_2_O_2_ produced by **Sn‐TMPyP** was assessed through bulk electrolysis experiments. Figure 8a shows the amperogram obtained during a CPE experiment, carried out by setting a potential for catalysis of 2.1 V versus RHE for 4 h in 0.2 mM **Sn‐TMPyP** and with a BDD plate with 2 cm^2^ surface area as working electrode. To determine the $FE_{H_{2}O_{2}}$ of the process, the amount of H_2_O_2_ produced was detected at various time intervals through titration with KMnO_4_ in acidic conditions (see Supporting Information for details).

Figure 8b shows that **Sn‐TMPyP** can generate hydrogen peroxide in a stable manner during a 4 h bulk electrolysis experiment with a remarkable $FE_{H_{2}O_{2}}$ of around 50% for the first 2h, which then slightly decreases to 38% after the third hour. The secondary reaction product is O_2_, likely obtained through the direct 4e‐WOR, as reported previously for similar systems [33, 34, 35, 36]. During this CPE experiment, 36 µmol of H_2_O_2_ were produced, corresponding to a productivity of about 6 µmol h^−1^ cm^−2^. The slight decrease in $FE_{H_{2}O_{2}}$ observed during the last 2 h of bulk electrolysis may be attributed to catalyst adsorption on the electrode or its degradation. This is supported by the comparison of CVs recorded before and after bulk electrolysis, which shows a reduction in the maximum catalytic current by 0.74 mA (Figure S8).

**Cu(tmpa)** catalyses the reduction of O_2_ in a stepwise manner with H_2_O_2_ as an obligatory intermediate, wherein the 2e‐ORR is faster than HPRR leading to high $FE_{H_{2}O_{2}}$ provided that mass transport is favorable [27, 28]. In bulk electrolysis experiments with an RDE [30], flow cells [72], and gas diffusion electrodes [73] wherein mass flow is optimal $FE_{H_{2}O_{2}}$ values above 50% can be obtained.

**FIGURE 9:**
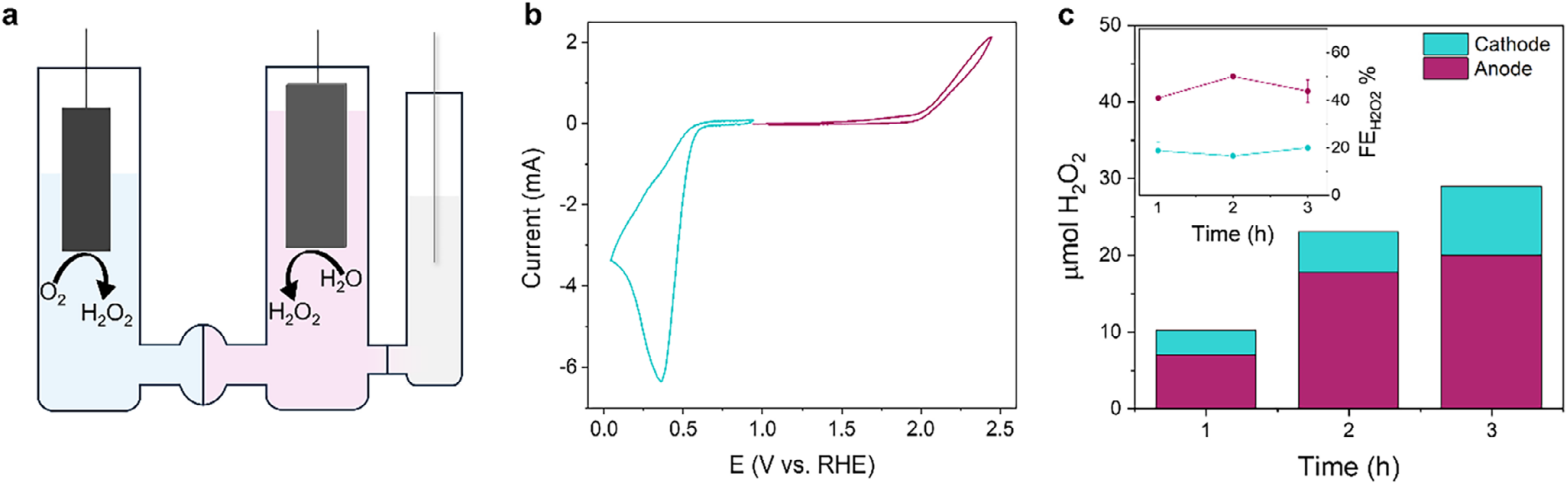
(a) Schematic representation of the H‐cell setup for convergent paired electrolysis, (b) Cathodic and anodic CV recorded in 0.2 mM **Sn‐TMPyP** under Ar at the anode and 0.2 mM **Cu(tmpa)** under O_2_ at the cathode in 0.1 M carbonate buffer at pH 9.2 (c) Faradaic efficiency for H_2_O_2_ (inset) and total µmol of H_2_O_2_ produced during 3 h CPE at 2.15 V vs. RHE with 0.2 mM **Sn‐TMPyP** under Ar at the anode and 0.2 mM **Cu(tmpa)** under O_2_ at the cathode in 0.1 M carbonate buffer at pH 9.2.

Water oxidation to H_2_O_2_ mediated by **Sn‐TMPyP** has been coupled to that of **Cu(tmpa)** as catalyst for the 2e^−^‐ORR to produce hydrogen peroxide on the two electrodes of an H‐cell, separated by a Forblue SELEMION AHO anionic exchange membrane (Figure 9a). A 0.1 M carbonate buffer at pH 9.2 has been employed as electrolyte to achieve sufficient catalytic activity of **Sn‐TMPyP**. Although the optimum pH window for **Cu(tmpa)** lies between 5 and 7, still a very satisfactory activity for the 2e^−^‐ORR with a turnover frequency of 1.2 × 10^6^ s^−1^ can be obtained at pH 9 [30]. Moreover, cell design required the use of stationary instead of rotating electrodes (BDD as anode and carbon paper as cathode). This caused restriction in oxygen gas diffusion and H_2_O_2_ accumulation near the electrode surface, globally leading to a reduced selectivity for 2e^−^‐ORR. The paired electrolysis tests were performed maintaining a 0.2 mM concentration of both **Cu(tmpa)** and **Sn‐TMPyP**, while the electrode surface area of both electrodes was 2 cm^2^. Preliminary CV tests under these conditions revealed larger catalytic currents at the cathode, owing to the higher ORR activity of **Cu(tmpa),** even in this electrolyte (Figure 9b). In order to maintain a counter electrode potential within the catalytic range of interest, the BDD anode was set as working electrode while the carbon paper cathode was set as the counter electrode. CPE experiments were performed for 3 h at 2.15 V versus RHE at the working electrode and the amount of H_2_O_2_ produced at the anode and cathode was quantified every hour by KMnO_4_ titration. The results obtained are reported in Figure 9c.

During CPE, the current remained stable at 0.9 mA for 120 min and started to decrease in the last hour, reaching a final value of 0.45 mA (Figure S9). This system produced notable amounts of H_2_O_2_ at an overpotential of 570 mV, divided over the cathode and the anode. The Faradaic efficiency for H_2_O_2_ over 3 h ranged from 15% to 19% at the cathode, likely due to the high local concentration of H_2_O_2_ near of the electrode surface and to the limited supply of O_2_, which restrained the selectivity of **Cu(tmpa)**. Nevertheless, **Cu(tmpa)** achieved an H_2_O_2_ production rate of 1.6 µmol h^−1^ cm^−2^. At the anode, the $FE_{H_{2}O_{2}}$ was 40%–50% for all the 3 h of CPE, with a production rate of 3.5 µmol h^−1^ cm^−2^. This last value is slightly lower than obtained when the same experiment was performed in a bulk electrolysis cell, probably due to compromised stirring inside the H‐cell compartment. The stability of the **Cu(tmpa)** and **Sn‐TMPyP** catalysts was assessed by recording CVs at the cathode and at the anode before and after CPE (Figure S10). The CV of **Cu(tmpa)** after 3 h electrolysis reveals a slight anodic shift of the potential for ORR and an increase in current, which likely originates from the presence of high H_2_O_2_ concentrations, leading to a significant increase of HPRR activity compared to the initial condition of the bulk electrolysis experiment. At the anode, the cyclic voltammogram recorded after CPE differs significantly from that obtained before the bulk electrolysis experiment. Specifically, the catalytic wave shifted to more cathodic values while the peak current decreased. To investigate whether the change of the voltammograms of the two catalysts is due to a variation of catalyst concentration, UV–vis spectra of **Cu(tmpa)** and **Sn‐TMPyP** were recorded before and after CPE (Figure S11). For **Cu(tmpa)**, the UV–vis spectra did not indicate a decrease in catalyst concentration over the 3 h electrolysis experiment. Only an increase in the absorbance at 250 nm is observed, which is attributed to the presence of H_2_O_2_ in solution [30]. On the other side, the UV–vis spectrum of **Sn‐TMPyP** revealed a relevant reduction of the absorption of the Soret band of the porphyrin at 450 nm after the electrolysis experiment, indicating that the concentration of the porphyrin in solution decreased during catalysis. The possible formation of catalytically active absorbed species during 3 h bulk electrolysis has been ruled out based on rinse tests of the BDD and carbon paper electrodes in a fresh electrolyte solution (Figure S12). At the cathode, the first voltammogram recorded in carbonate buffer revealed the presence of ORR current at 0.2 V versus RHE, which can be ascribed to the physical adsorption of **Cu(tmpa)** on the porous surface of the electrode as after 20 cycles this catalytic current diminished, approaching the blank current values. Analogously, a CV of the BDD electrode in fresh carbonate buffer showed catalytic current in the first cycle at 2.09 V versus RHE, which disappeared after 20 scans. From these experiments, we can conclude that both catalysts are active as molecular species in solution for 2e^−^‐ORR and 2e^−^‐WOR. Specifically, **Cu(tmpa)** is stable for over 3 h electrolysis and limitedly adsorbed on the carbon paper electrode surface. On the other side, **Sn‐TMPyP** undergoes a degradation process after 2 h of electrolysis thus explaining the progressive decrease of the catalytic current after 120 min. The UV–vis spectra recorded before and after bulk electrolysis suggest that the porphyrin is deposited on the electrode surface as a catalytically inactive species. A similar behavior has been observed on the 2e‐WOR catalysts **Si‐TPyP** and **Zn‐TMPyP** following bulk electrolysis [35, 36].

To achieve a higher $FE_{H_{2}O_{2}}$ in the cathode compartment we resided to cobalt 2e‐ORR catalysts [13, 14]. These cobalt systems are known to reduce O_2_ to H_2_O_2_ via cobalt superoxide and hydroperoxide intermediates [15], but are rarely studied in aqueous solutions and by bulk electrolysis experiments. Based on a comparison study by the Stahl group in organic solvent, we considered five cobalt catalysts [13, 14]. From preliminary studies we excluded **Co‐OEP**, **Co(salen)**, and **Co‐TMPyP** due to very low performance in test experiments, and on basis of RDE CPE experiments (see Supporting Information) we pinpointed **Co(Salphen–OH)** as the more competent catalyst over **CoTPP** and **Co(Salphen–OMe)**: 2e^−^‐ORR by **Co(Salphen‐OH)** is characterized by an onset near 0.8 V versus RHE, a catalytic current that is mass transport limited in RDE experiments, a Tafel slope of 66 mV/dec, and a $FE_{H_{2}O_{2}}$ above 80%. Paired electrolysis employing **Sn‐TMPyP** and **Co(Salphen–OH)** resulted in an overall efficiency >90% over both compartments after 1h.

Our work shows the production of H_2_O_2_ by paired electrolysis where **Sn‐TMPyP** is used as a 2e^−^‐WOR catalyst and **Cu(tmpa)** or **Co(Salphen‐OH)** as a 2e^−^‐ORR catalyst, operating in a carbonate buffer wherein both catalytic reactions are compatible. The $FE_{H_{2}O_{2}}$ of the 2e^−^‐ORR in the paired electrolysis H‐cell is below that of RDE studies, presumably due to poor mass transport of O_2_ and H_2_O_2_ [30], while the stability of **Co(Salphen‐OH)** and **Sn‐TMPyP** upon the build‐up of H_2_O_2_ limits the operating lifetime. These findings illustrate the need to evaluate homogeneous catalysts in realistic systems and during the build‐up of H_2_O_2_ concentrations. It is important to note that 2e^−^‐WOR demands a high potential (> 1.8 V versus RHE), which is notably higher than that required for 4e^−^WOR (> 1.23 V versus RHE). Under these highly oxidizing conditions, **Sn‐TMPyP** demonstrated remarkable longevity during CPE experiments and achieved a $\;FE_{H_{2}O_{2}}$ that is among the highest ever reported for molecular catalysts for this reaction (Table 1).

**TABLE 1:** Comparison of 2e WOR catalysts.

Catalyst	Electrolyte	pH	η (mV)	TOF (s^−1^)	F.E.
**Al‐TMPyP** [33]	0.1 M sodium acetate	12.5	97	2 × 10^4^	59%*
**Zn‐TMPyP** [36]	0.1 M sodium sulfate	12.3	67	96.4	34%*
**Si‐TPyP** [35]	AcN/H_2_O with 1 mM sodium carbonate				60%*
**Sn‐TMPyP**	0.1 M carbonate buffer	9.2	240	1 × 10^3^	50%

*$FE_{H_{2}O_{2}}$ was calculated based on the total concentration of free H_2_O_2_ and M‐OOH in solution.

## 3 Conclusion

The current synthetic methods used to produce hydrogen peroxide – an important bulk chemical employed as a green oxidant and as an energy carrier in certain types of fuel cells – have critical drawbacks, particularly due to energy‐intensive purification processes associated with the synthesis process and the safety hazards involved with handling and shipping concentrated peroxide. Electrosynthesis of H_2_O_2_, especially if conducted in aqueous solution, using electricity coming from a renewable source, represents a highly desired and sustainable alternative. The main obstacle for developing an electrosynthetic apparatus for the selective and efficient electrosynthesis of H_2_O_2_ stems from developing proper cathodic and anodic catalytic systems. In our paper we succeeded in assembling a cell for the paired electrosynthesis of H_2_O_2_, mediated by homogeneous catalysts based on earth‐abundant metals on both electrodes. It exhibits remarkable activity with a key role played by the **Sn‐TMPyP** catalyst for the 2e^−^‐WOR in combination with carbonate buffer as electrolyte, boosting the catalytic performance to a TOF of 1.1 × 10^3^ s^−1^ and increasing the H_2_O_2_ productivity during CPE experiments from 0.13 µmol h^−1^ cm^−2^ in 0.1 M Na_2_SO_4_ at pH 8.5 to 6 µmol h^−1^ cm^−2^ (0.1 M carbonate buffer at pH 9.2). The Faradaic efficiency towards the desired product was more than doubled in comparison to previous studies, reaching 50% during bulk electrolysis experiments.

The use of molecular catalysts in both electrode compartments allowed for an in‐depth mechanistic description that can be used to improve performance. At the same time, the results obtained are already so promising that we can conclude that our study paves the way to the development of an apparatus for a large‐scale production of hydrogen peroxide based single‐site homogeneous catalysts.

## Supporting Information

The authors have cited additional references within the Supporting Information [1, 2, 3, 4, 5, 6, 7, 8, 9, 10, 11, 12].

## Conflicts of Interest

The authors declare no conflicts of interest.

## Supporting information




**Supporting File 1**: anie71856‐sup‐0002‐SuppMat.docx.

**Supporting File 2**: anie71856‐sup‐0001‐Data.zip.

## Data Availability

The data that support the findings of this study are available in the supplementary material of this article.

## References

[1] S. Fukuzumi , Y. Yamada , and K. D. Karlin , “Hydrogen Peroxide as a Sustainable Energy Carrier: Electrocatalytic Production of Hydrogen Peroxide and the Fuel Cell,” Electrochimica Acta 82 (2012): 493–511, 10.1016/j.electacta.2012.03.132. 23457415 PMC3584454

[2] L. An , T. Zhao , X. Yan , X. Zhou , and P. Tan , “The Dual Role of Hydrogen Peroxide in Fuel Cells,” Science Bulletin 60 (2015): 55–64, 10.1007/s11434-014-0694-7.

[3] L. An , T. S. Zhao , and J. B. Xu , “A bi‐functional Cathode Structure for Alkaline‐acid Direct Ethanol Fuel Cells,” International Journal of Hydrogen Energy 36 (2011): 13089–13095, 10.1016/j.ijhydene.2011.07.025.

[4] J. M. Campos‐Martin , G. Blanco‐Brieva , and J. L. G. Fierro , “Hydrogen Peroxide Synthesis: An Outlook Beyond the Anthraquinone Process,” Angewandte Chemie International Edition 45 (2006): 6962–6984, 10.1002/anie.200503779. 17039551

[5] Y. Li , Y. Zhang , G. Xia , J. Zhan , G. Yu , and Y. Wang , “Evaluation of the Technoeconomic Feasibility of Electrochemical Hydrogen Peroxide Production for Decentralized Water Treatment,” Frontiers of Environmental Science & Engineering 15 (2020): 1, 10.1007/s11783-020-1293-2.

[6] Y. Sun , L. Han , and P. Strasser , “A Comparative Perspective of Electrochemical and Photochemical Approaches for Catalytic H_2_ O_2_ Production,” Chemical Society Reviews 49 (2020): 6605–6631, 10.1039/D0CS00458H. 32760937

[7] N. Wang , S. Ma , P. Zuo , J. Duan , and B. Hou , “Recent Progress of Electrochemical Production of Hydrogen Peroxide by Two‐Electron Oxygen Reduction Reaction,” Advanced Science 8 (2021): 2100076, 10.1002/advs.202100076. 34047062 PMC8336511

[8] Y. Wang , G. I. N. Waterhouse , L. Shang , and T. Zhang , “Electrocatalytic Oxygen Reduction to Hydrogen Peroxide: From Homogeneous to Heterogeneous Electrocatalysis,” Advanced Energy Materials 11 (2021): 2003323, 10.1002/aenm.202003323.

[9] S. C. Perry , D. Pangotra , L. Vieira , et al., “Electrochemical Synthesis of Hydrogen Peroxide From Water and Oxygen,” Nature Reviews Chemistry 3 (2019): 442–458, 10.1038/s41570-019-0110-6.

[10] W. Zhang , W. Lai , and R. Cao , “Energy‐Related Small Molecule Activation Reactions: Oxygen Reduction and Hydrogen and Oxygen Evolution Reactions Catalyzed by Porphyrin‐ and Corrole‐Based Systems,” Chemical Reviews 117 (2017): 3717–3797, 10.1021/acs.chemrev.6b00299. 28222601

[11] K. Mase , K. Ohkubo , and S. Fukuzumi , “Efficient Two‐Electron Reduction of Dioxygen to Hydrogen Peroxide With One‐Electron Reductants With a Small Overpotential Catalyzed by a Cobalt Chlorin Complex,” Journal of the American Chemical Society 135 (2013): 2800–2808, 10.1021/ja312199h. 23343346

[12] T. Honda , T. Kojima , and S. Fukuzumi , “Proton‐Coupled Electron‐Transfer Reduction of Dioxygen Catalyzed by a Saddle‐Distorted Cobalt Phthalocyanine,” Journal of the American Chemical Society 134 (2012): 4196–4206, 10.1021/ja209978q. 22299646

[13] Y. H. Wang , M. L. Pegis , J. M. Mayer , and S. S. Stahl , “Molecular Cobalt Catalysts for O_2_ Reduction: Low‐Overpotential Production of H_2_ O_2_ and Comparison With Iron‐Based Catalysts,” Journal of the American Chemical Society 139 (2017): 16458–16461, 10.1021/jacs.7b09089. 29039921

[14] Y. H. Wang , B. Mondal , and S. S. Stahl , “Molecular Cobalt Catalysts for O_2_ Reduction to H_2_ O_2_: Benchmarking Catalyst Performance via Rate–Overpotential Correlations,” American Chemical Society Catalysis 10 (2020): 12031–12039, 10.1021/acscatal.0c02197.

[15] Y. H. Wang , Z. K. Goldsmith , P. E. Schneider , et al., “Kinetic and Mechanistic Characterization of Low‐Overpotential, H_2_ O_2_ ‐Selective Reduction of O_2_ Catalyzed by N_2_ O_2_ ‐Ligated Cobalt Complexes,” Journal of the American Chemical Society 140 (2018): 10890–10899, 10.1021/jacs.8b06394. 30060652

[16] J. Rosenthal and D. G. Nocera , “Role of Proton‐Coupled Electron Transfer in O–O Bond Activation,” Accounts of Chemical Research 40 (2007): 543–553, 10.1021/ar7000638. 17595052

[17] S. Amanullah , P. K. Das , S. Samanta , and A. Dey , “Tuning the Thermodynamic Onset Potential of Electrocatalytic O_2_ Reduction Reaction by Synthetic Iron–porphyrin Complexes,” Chemical Communications 51 (2015): 10010–10013, 10.1039/C5CC01938A. 26000662

[18] H. Maid , P. Böhm , S. M. Huber , et al., “Iron Catalysis for in Situ Regeneration of Oxidized Cofactors by Activation and Reduction of Molecular Oxygen: A Synthetic Metalloporphyrin as a Biomimetic NAD(P)H Oxidase,” Angewandte Chemie International Edition 50 (2011): 2397–2400, 10.1002/anie.201004101. 21351363

[19] E. N. Cook , D. A. Dickie , and C. W. Machan , “Catalytic Reduction of Dioxygen to Water by a Bioinspired Non‐Heme Iron Complex via a 2+2 Mechanism,” Journal of the American Chemical Society 143 (2021): 16411–16418, 10.1021/jacs.1c04572. 34606274

[20] L. Wang , M. Gennari , F. G. Cantú Reinhard , et al., “A Non‐Heme Diiron Complex for (Electro)Catalytic Reduction of Dioxygen: Tuning the Selectivity Through Electron Delivery,” Journal of the American Chemical Society 141 (2019): 8244–8253, 10.1021/jacs.9b02011. 31026148

[21] N. W. G. Smits , B. van Dijk , I. de Bruin , S. L. T. Groeneveld , M. A. Siegler , and D. G. H. Hetterscheid , “Influence of Ligand Denticity and Flexibility on the Molecular Copper Mediated Oxygen Reduction Reaction,” Inorganic Chemistry 59 (2020): 16398–16409, 10.1021/acs.inorgchem.0c02204. 33108871 PMC7672700

[22] N. W. G. Smits , D. Rademaker , A. I. Konovalov , M. A. Siegler , and D. G. H. Hetterscheid , “Influence of the Spatial Distribution of Copper Sites on the Selectivity of the Oxygen Reduction Reaction,” Dalton Transactions 51 (2022): 1206–1215, 10.1039/D1DT03296H. 34951437 PMC8763313

[23] H. Kotani , T. Yagi , T. Ishizuka , and T. Kojima , “Enhancement of 4‐electron O_2_ Reduction by a Cu( ii )–pyridylamine Complex via Protonation of a Pendant Pyridine in the Second Coordination Sphere in Water,” Chemical Communications 51 (2015): 13385–13388, 10.1039/C5CC03012A. 26207327

[24] M. A. Thorseth , C. E. Tornow , E. C. M. Tse , and A. A. Gewirth , “Cu Complexes That Catalyze the Oxygen Reduction Reaction,” Coordination Chemistry Reviews 257 (2013): 130–139, 10.1016/j.ccr.2012.03.033.

[25] T. Kuwana , M. Fujihira , K. Sunakawa , and T. Osa , “Catalytic Electroreduction of Molecular Oxygen Using Water Soluble Iron Porphyrin,” Journal of Electroanalytical Chemistry and Interfacial Electrochemistry 88 (1978): 299–303, 10.1016/S0022-0728(78)80278-2.

[26] M. L. Pegis , B. A. McKeown , N. Kumar , et al., “Homogenous Electrocatalytic Oxygen Reduction Rates Correlate With Reaction Overpotential in Acidic Organic Solutions,” ACS Central Science 2 (2016): 850–856, 10.1021/acscentsci.6b00261. 27924314 PMC5126711

[27] M. Langerman and D. G. H. Hetterscheid , “Fast Oxygen Reduction Catalyzed by a Copper(II) Tris(2‐pyridylmethyl)Amine Complex Through a Stepwise Mechanism,” Angewandte Chemie International Edition 58 (2019): 12974–12978, 10.1002/anie.201904075. 31339205

[28] M. Langerman and D. G. H. Hetterscheid , “Mechanistic Study of the Activation and the Electrocatalytic Reduction of Hydrogen Peroxide by Cu‐Tmpa in Neutral Aqueous Solution,” ChemElectroChem 8 (2021): 2783–2791, 10.1002/celc.202100436. 34589379 PMC8453753

[29] M. Langerman , M. van Dorth , and D. G. H. Hetterscheid , “Dioxygen Reduction in Acetonitrile With Copper Pyridylalkylamine Complexes: The Influence of Acid Strength on the Catalytic Performance,” European Journal of Inorganic Chemistry 26 (2023): e202300218, 10.1002/ejic.202300218.

[30] P. H. van Langevelde and D. G. H. Hetterscheid , “Selective Electrochemical H2O2 Production by a Molecular Copper Catalyst: A Crucial Relation Between Reaction Rate and Mass Transport,” Chem Catalysis 4 (2024): 101069, 10.1016/j.checat.2024.101069.

[31] S. Mavrikis , S. C. Perry , P. K. Leung , L. Wang , and C. Ponce de León , “Recent Advances in Electrochemical Water Oxidation to Produce Hydrogen Peroxide: A Mechanistic Perspective,” ACS Sustainable Chemistry & Engineering 9 (2021): 76–91, 10.1021/acssuschemeng.0c07263.

[32] X. Shi , S. Back , T. M. Gill , S. Siahrostami , and X. Zheng , “Electrochemical Synthesis of H2O2 by Two‐Electron Water Oxidation Reaction,” Chemistry 7 (2021): 38–63, 10.1016/j.chempr.2020.09.013.

[33] F. Kuttassery , S. Mathew , S. Sagawa , et al., “One Electron‐Initiated Two‐Electron Oxidation of Water by Aluminum Porphyrins With Earth's Most Abundant Metal,” Chemsuschem 10 (2017): 1909–1915, 10.1002/cssc.201700322. 28322007

[34] Y. Ohsaki , A. Thomas , F. Kuttassery , et al., “Two‐electron Oxidation of Water to Form Hydrogen Peroxide Initiated by One‐electron Oxidation of Tin (IV)‐porphyrins,” Journal of Photochemistry and Photobiology A: Chemistry 401 (2020): 112732, 10.1016/j.jphotochem.2020.112732.

[35] S. N. Remello , F. Kuttassery , S. Mathew , et al., “Two‐electron Oxidation of Water to Form Hydrogen Peroxide Catalysed by Silicon‐porphyrins,” Sustainable Energy Fuels 2 (2018): 1966–1973, 10.1039/C8SE00102B.

[36] A. Sebastian , S. N. Remello , F. Kuttassery , et al., “Protolytic Behavior of Water‐soluble Zinc(II) Porphyrin and the Electrocatalytic Two‐electron Water Oxidation to Form Hydrogen Peroxide,” Journal of Photochemistry and Photobiology A: Chemistry 400 (2020): 112619, 10.1016/j.jphotochem.2020.112619.

[37] J. Baek , Q. Jin , N. S. Johnson , et al., “Discovery of LaAlO3 as an Efficient Catalyst for Two‐electron Water Electrolysis towards Hydrogen Peroxide,” Nature Communications 13 (2022): 7256, 10.1038/s41467-022-34884-4. PMC9700689 36433962

[38] A. Thomas , F. Kuttassery , S. Mathew , et al., “Protolytic Behavior of Axially Coordinated Hydroxy Groups of Tin(IV) Porphyrins as Promising Molecular Catalysts for Water Oxidation,” Journal of Photochemistry and Photobiology A: Chemistry 358 (2018): 402–410, 10.1016/j.jphotochem.2017.09.053.

[39] C. Trotta , P. Dahiya , L. Baldinelli , et al., “A Cobalt Molecular Catalyst for Hydrogen Evolution Reaction With Remarkable Activity in Phosphate Buffered Water Solution,” Catalysis Science & Technology 14 (2024): 3699–3706, 10.1039/D4CY00209A.

[40] L. Fan , X. Bai , C. Xia , et al., “CO2/carbonate‐mediated Electrochemical Water Oxidation to Hydrogen Peroxide,” Nature Communications 13 (2022): 2668, 10.1038/s41467-022-30251-5. PMC9106728 35562346

[41] H. Ni , T. Yang , X. Peng , H. Zhang , and A. Kong , “Membrane‐Free Electrolysis Production of Hydrogen Peroxide on Low‐Cost Metal‐Free Electrocatalysts for Dye Degradation,” Chemistry – A European Journal 30 (2024): e202403279, 10.1002/chem.202403279. 39501718

[42] T. Schanz , M. Stöckl , B. O. Burek , D. Holtmann , and J. Z. Bloh , “Combined Anodic and Cathodic Peroxide Production in an Undivided Carbonate/Bicarbonate Electrolyte With 144% Combined Current Efficiency,” Frontiers in Catalysis 4 (2024): 1353746, 10.3389/fctls.2024.1353746.

[43] Y. Sun , K. Fan , J. Li , et al., “Boosting Electrochemical Oxygen Reduction to Hydrogen Peroxide Coupled With Organic Oxidation,” Nature Communications 15 (2024): 6098, 10.1038/s41467-024-50446-2. PMC11271547 39030230

[44] C. Kim , S. O. Park , S. K. Kwak , Z. Xia , G. Kim , and L. Dai , “Concurrent Oxygen Reduction and Water Oxidation at High Ionic Strength for Scalable Electrosynthesis of Hydrogen Peroxide,” Nature Communications 14 (2023): 5822, 10.1038/s41467-023-41397-1. PMC10509222 37726271

[45] X. Huang , M. Song , J. Zhang , T. Shen , G. Luo , and D. Wang , “Recent Advances of Electrocatalyst and Cell Design for Hydrogen Peroxide Production,” Nano‐Micro Letters 15 (2023): 86, 10.1007/s40820-023-01044-2. 37029260 PMC10082148

[46] S. Wang , Z. Xie , D. Zhu , et al., “Efficient Photocatalytic Production of Hydrogen Peroxide Using Dispersible and Photoactive Porous Polymers,” Nature Communications 14 (2023): 6891, 10.1038/s41467-023-42720-6. PMC10613291 37898686

[47] C. He , Z. Luo , L. Zhang , Q. Zhang , C. He , and X. Ren , “Progress and Challenges for Electrocatalytic Production of Hydrogen Peroxide,” Applied Catalysis A: General 681 (2024): 119803, 10.1016/j.apcata.2024.119803.

[48] A. Thomas , F. Kuttassery , S. N. Remello , et al., “Facile Synthesis of Water‐Soluble Cationic Tin(IV) Porphyrins and Water‐Insoluble Tin(IV) Porphyrins in Water at Ambient Temperature,” Bulletin of the Chemical Society of Japan 89 (2016): 902–904, 10.1246/bcsj.20160091.

[49] See Supporting info for experimental details. The CCDC deposition number, 2474879 contain the supplementary crystallographic data for this paper. These data are provided free of charge by the joint Cambridge Crystallographic Data Centre and Fachinformationszentrum Karlsruhe Access Structures service.

[50] T. Lang , E. Graf , N. Kyritsakas , and M. W. Hosseini , “Open and Closed States of a Porphyrin Based Molecular Turnstile,” Dalton Transactions 40 (2011): 3517, 10.1039/c1dt00004g. 21365109

[51] Y. Liu , Y. Han , Z. Zhang , et al., “Low Overpotential Water Oxidation at Neutral pH Catalyzed by a Copper( ii ) Porphyrin,” Chemical Science 10 (2019): 2613–2622, 10.1039/C8SC04529A. 30996977 PMC6419937

[52] A. Macchioni , G. Ciancaleoni , C. Zuccaccia , and D. Zuccaccia , “Determining Accurate Molecular Sizes in Solution Through NMR Diffusion Spectroscopy,” Chemical Society Reviews 37 (2008): 479–489, 10.1039/B615067P. 18224258

[53] F. Zaccaria , C. Zuccaccia , R. Cipullo , and A. Macchioni , “Extraction of Reliable Molecular Information From Diffusion NMR Spectroscopy: Hydrodynamic Volume or Molecular Mass?,” Chemistry – A European Journal 25 (2019): 9930–9937, 10.1002/chem.201900812. 30998838

[54] C. Zuccaccia , G. Bellachioma , G. Cardaci , and A. Macchioni , “Self‐Diffusion Coefficients of Transition‐Metal Complex Ions, Ion Pairs, and Higher Aggregates by Pulsed Field Gradient Spin−Echo NMR Measurements,” Organometallics 19 (2000): 4663–4665, 10.1021/om000574c.

[55] G. Bellachioma , G. Ciancaleoni , C. Zuccaccia , D. Zuccaccia , and A. Macchioni , “NMR Investigation of Non‐covalent Aggregation of Coordination Compounds Ranging From Dimers and Ion Pairs Up to Nano‐aggregates,” Coordination Chemistry Reviews 252 (2008): 2224–2238, 10.1016/j.ccr.2007.12.016.

[56] F. Zaccaria , A. Macchioni , and C. Zuccaccia , “Accurate Determination of Molecular Sizes of a Solute in Water from Its Translational Self‐Diffusion Coefficient,” Chemistry–Methods 5 (2025): e202400063, 10.1002/cmtd.202400063.

[57] E. Van Caemelbecke , A. Derbin , P. Hambright , et al., “Electrochemistry of [(TMpyP)M^II^ ]^4+^ (X^−^)_4_ (X^−^=Cl^−^or BPh^−^) and [(TMpyP)M^III^ Cl]^4+^ (Cl^−^ )_4_ in N , N ‐Dimethylformamide Where M Is One of 15 Different Metal Ions,” Inorganic Chemistry 44 (2005): 3789–3798, 10.1021/ic048820q. 15907103

[58] Z. Ou , W. E , W. Zhu , et al., “Effect of Axial Ligands and Macrocyclic Structure on Redox Potentials and Electron‐Transfer Mechanisms of Sn(IV) Porphyrins,” Inorganic Chemistry 46 (2007): 10840–10849, 10.1021/ic7016165. 17994732

[59] S. Baral , P. Hambright , and P. Neta , “One‐ and Two‐electron Reduction of Aluminum and Tin Pyridylporphyrins. A Kinetic Spectrophotometric Study,” Journal of Physical Chemistry 88 (1984): 1595–1600, 10.1021/j150652a031.

[60] C. P. Andrieux , J. M. Dumas‐Bouchiat , and J. M. Savéant , “Homogeneous Redox Catalysis of Electrochemical Reactions,” Journal of Electroanalytical Chemistry and Interfacial Electrochemistry 113 (1980): 1–18, 10.1016/S0022-0728(80)80507-9.

[61] M. W. Hsiao , R. R. Adžić , and E. B. Yeager , “Electrochemical Oxidation of Glucose on Single Crystal and Polycrystalline Gold Surfaces in Phosphate Buffer,” Journal of the Electrochemical Society 143 (1996): 759–767, 10.1149/1.1836536.

[62] F. Kuttassery , A. Sebastian , S. Mathew , H. Tachibana , and H. Inoue , “Promotive Effect of Bicarbonate Ion on Two‐Electron Water Oxidation to Form H_2_ O_2_ Catalyzed by Aluminum Porphyrins,” Chemsuschem 12 (2019): 1939–1948, 10.1002/cssc.201900560. 30963704

[63] S. Mavrikis , M. Göltz , S. C. Perry , et al., “Effective Hydrogen Peroxide Production From Electrochemical Water Oxidation,” ACS Energy Letters 6 (2021): 2369–2377, 10.1021/acsenergylett.1c00904.

[64] S. Mavrikis , M. Göltz , S. Rosiwal , L. Wang , and C. Ponce de León , “Carbonate‐Induced Electrosynthesis of Hydrogen Peroxide via Two‐Electron Water Oxidation,” Chemsuschem 15 (2022): e202102137, 10.1002/cssc.202102137. 34935302

[65] Y. F. Miao , W. Y. Wu , H. L. Xie , Z. H. Yang , T. Q. Huang , and H. T. Liu , “The Pivotal Role of Carbonates in Electrocatalytic Water Oxidation for Hydrogen Peroxide Production: Performance, Mechanisms, and Future Perspectives,” Carbon Neutralization 4 (2025): e70074, 10.1002/cnl2.70074.

[66] C. Trotta , G. Menendez Rodriguez , C. Zuccaccia , and A. Macchioni , “Electrochemical NADH Regeneration Mediated by Pyridine Amidate Iridium Complexes Interconverting 1,4‐ and 1,6‐NADH,” ACS Catalysis 14 (2024): 10334–10343, 10.1021/acscatal.4c02548.

[67] R. M. Bullock , A. M. Appel , and M. L. Helm , “Production of Hydrogen by Electrocatalysis: Making the H–H Bond by Combining Protons and Hydrides,” Chemical Communications 50 (2014): 3125–3143, 10.1039/C3CC46135A. 24448464

[68] D. den Boer , A. I. Konovalov , M. A. Siegler , and D. G. H. Hetterscheid , “Unusual Water Oxidation Mechanism via a Redox‐Active Copper Polypyridyl Complex,” Inorganic Chemistry 62 (2023): 5303–5314, 10.1021/acs.inorgchem.3c00477. 36989161 PMC10091478

[69] Z. Chen , J. J. Concepcion , X. Hu , W. Yang , P. G. Hoertz , and T. J. Meyer , “Concerted O Atom–proton Transfer in the O—O Bond Forming Step in Water Oxidation,” Proceedings of the National Academy of Sciences 107 (2010): 7225–7229, 10.1073/pnas.1001132107. PMC2867729 20360565

[70] Z. Wen , B. Fan , Z. Mao , et al., “Bicarbonate‐Promoted Hydrogen Peroxide Activation for Selective Electrochemical Oxidation of Small Molecules,” ACS Energy Letters 10 (2025): 6262–6267, 10.1021/acsenergylett.5c03021.

[71] D. E. Richardson , H. Yao , K. M. Frank , and D. A. Bennett , “Equilibria, Kinetics, and Mechanism in the Bicarbonate Activation of Hydrogen Peroxide: Oxidation of Sulfides by Peroxymonocarbonate,” Journal of the American Chemical Society 122 (2000): 1729–1739, 10.1021/ja9927467.

[72] N. E. G. Ligthart , P. H. Van Langevelde , J. T. Padding , D. G. H. Hetterscheid , and D. A. Vermaas , “20‐Fold Increased Limiting Currents in Oxygen Reduction With Cu‐tmpa by Replacing Flow‐By With Flow‐Through Electrodes,” ACS Sustainable Chemistry & Engineering 12 (2024): 12909–12918, 10.1021/acssuschemeng.4c03919. 39211382 PMC11351704

[73] P. H. Van Langevelde , N. E. G. Ligthart , P. G. J. Van Duren , D. A. Vermaas , and D. G. H. Hetterscheid , ACS Electrochemistry 1 (2025): 2326–2337. 10.1021/acselectrochem.5c00179 PMC12598710 41220610

