# Supplementary material for: A Homogeneously Catalyzed Paired Electrolytic Cell for Hydrogen Peroxide Production
Source: Angew Chem Int Ed Engl. 2026 Mar 23;65(19):e24811. doi: 10.1002/anie.202524811 (PMC13134611; doi:10.1002/anie.202524811)
Supplement: Supplementary file 1 — Supporting File 1: anie71856‐sup‐0002‐SuppMat.docx. [file ANIE-65-e24811-s001.docx]

## Supporting information

**General**

All chemicals and solvents were purchased from commercial suppliers and used without any further purification. 5,10,15,20-Tetrakis(1-methyl-4-pyridinio)porphyrin, tetra(p-toluenesulfonate), SnCl_2_ and Nafion© (5 wt. % in alcohols) were purchased by Sigma-Aldrich. Cobalt tetraphenylporphyrin (98%) and cobalt octaethylporphyrin (98%) were purchased from PorphyChem. Carbon black (acetylene, 50% compressed, 99.9+%) and [Co(Salen)] (96%) were purchased from Alfa Aesar. Reaction grade acetone (99.5%) was purchased from Boom B.V. The electrolyte solutions were prepared from high purity salts and acids, Na_2_HPO_4_ (≥99.999% TraceSELECT, Honeywell Fluka), NaH_2_PO_4_ (99.99% Suprapur, Merck), NaOH·H_2_O (≥99.9995% TraceSELECT, Honeywell Fluka), H_2_SO_4_ (96% in H_2_O, Merck, Suprapur), H_3_BO_3_ (99.999% trace metals basis, Sigma-Aldrich), KHCO_3_ (>99.5 %, Sigma- Aldrich), K_2_CO_3_ (99.995 %, Sigma- Aldrich). The O_2_, H_2_, and Ar gasses used in electrochemical measurements were supplied by Linde. UV-Vis spectra were recorded on a Varian Cary 50 spectrophotometer from Agilent. [Cu(tmpa)MeCN](OTf)_2_, [Sn(TMPyP)Cl_2_]Cl_2_, [Co(Salphen-OH)] and [Co-(Salphen-OMe)] were synthesized as previously reported.^[1-3]^ [Co(TMPyP)(H_2_O)2](PF_6_)_5_ was synthesized as reported previously, yet the final product was obtained as an aqua complex by precipitation with aqueous ammonium hexafluorophosphate.^[4]^

**Single Crystal X-Ray Crystallography**

All reflection intensities were measured at 223.00(10) K using a Rigaku XtaLAB Synergy R (equipped with a rotating-anode X-ray source and HyPix-6000HE detector) with Cu *K*α radiation (λ = 1.54178 Å) under the program CrysAlisPro (Version CrysAlisPro 1.171.42.49, Rigaku OD, 2022). The same program was used to refine the cell dimensions and for data reduction. The structure was solved with the program SHELXT-2018/2^[5]^ and was refined on *F^2^* with SHELXL-2019/3.^[5]^ Crystals were initially deposited on a microscope slide in some Parabar 10312 and were cooled under a cold N_2_(g) stream in order to prevent any decomposition of the crystals. One single crystal was then quickly picked and mounted on the diffractometer while being flash-cooled at 223 K. Analytical numeric absorption correction using a multifaceted crystal model was applied using CrysAlisPro. The temperature of the data collection was controlled using the system Cryostream 1000 from Oxford Cryosystems. The H atoms were placed at calculated positions using the instructions AFIX 43, AFIX 137 or AFIX 143 with isotropic displacement parameters having values 1.2 or 1.5 *U*_eq_ of the attached C or O atoms.

* Attempts to collect data at 110, 173 and 193 K were made, but significant crystal damage always occurred, which is possibly due to a destructive solid-solid phase transition.

At 223 K, the crystal remained stable, and diffraction was of good quality. The structure is mostly ordered. The asymmetric unit contains three ordered lattice MeOH solvent molecules, and two of these molecules are partially occupied (with occupancy factors of 0.854(5) and 0.786(6)). At 223 K, the crystal remained stable, and diffraction was of good quality.  The structure is mostly ordered.  The asymmetric unit contains ½ of the Sn^IV^ complex (as it is found located at one site of inversion symmetry), 2 Cl^–^ counterions, three ordered lattice MeOH solvent molecules (two of these molecules are found to be partially occupied with occupancy factors of 0.854(5) and 0.786(6)). The asymmetric unit also contains some very disordered lattice solvent molecules (and most likely partially occupied), and their contribution was removed from the final refinement using the SQUEEZE procedure in Platon.^[4]^

**Electrochemical CV measurements**

All electrochemical measurements were carried out using PGSTAT 12, 204, and 128N potentiostats in combination with the NOVA 2 software package. The glassware used for electrochemical measurements was regularly cleaned by soaking in a solution of KMnO_4_ (1 g/L) in 0.5 M H_2_SO_4_ overnight. The next day, the glassware was rinsed and soaked in a diluted solution of H_2_O_2_ and H_2_SO_4_ for 30 – 60 minutes. Thereafter, the glassware was rinsed and boiled three times in Milli-Q. Prior to each experiment all glassware was boiled in Milli-Q water to remove any leftover salts. Unless stated otherwise, all CV measurements in still solution were carried out in a custom-made three-electrode cell with a volume of 10 mL In all experiments involving **Sn-TMPyP** a boron doped diamond (BDD) from Windsor Scientific Ltd (A = 0.07 cm^2^) was used as working electrode (WE). Before every measurement the BDD WE was polished on a LaboPol-30 polishing machine from Struers for 2 minutes with silica suspension (0.04 µm) on Dur-type polishing cloths. Afterwards, the BDD was sonicated for 15 minutes in Milli-Q water to remove any leftover polishing solution and electropolished in 0.1 M H_2_SO_4_ by performing 200 CVs between -1.26 V and + 1.99 V vs. Ag/AgCl at 1 Vs^-1^. A large gold wire was used as a counter electrode (CE) in all experiments, which was flame-annealed and rinsed with water prior to each experiment. For all the experiments a double-junction Ag/AgCl reference electrode filled with 3 M KCl (Metrohm) was used. Before the start of every experiment, the electrolyte solutions were saturated with Ar or O_2_ for at least 15 minutes. During the experiments a flow of argon or oxygen over the solution was maintained. All CV measurements were carried out at scan rate of 100 mVs^-1^, unless stated otherwise.

**CPE experiments**

CPE experiments for the production of hydrogen peroxide with **Sn-TMPyP** were carried out in a custom-made small three-electrode RDE cell with a minimum volume of 40 mL. The WE used was a BDD/Si Electrode from NeoCoat coated with p-doped BDD layer with a 3 mm thickness. The electrode was taped with Teflon tape to have an active surface area of 2 cm^2^. This electrode was polished according to literature procedures^[7–9]^ performing a galvanostatic chronopotentiometry at 1 A cm^-2^ for 1 h in an aqueous solution of 3 M acetic acid and 1 M H_2_SO_4_ at 40 °C using a high surface area Pt grid as CE.

**Preparation of Co-dropcasts**

The catalytic inks were prepared by suspending 1 µmole of cobalt catalyst (**Co(Salphen-OH)**, **Co(Salphen-OMe)** or **Co-TPP**), Nafion (with a pipette set to 4 µL) and 3.6 mg carbon black in 8 mL of acetone. To dropcast the ink in a well-defined manner a spin coating machine (Chemat Technology spin-coater KW-4a) was used. A carbon paper (Ion Power GmbH) electrode cut with a scalpel to a circle with an area of 2 cm^2^. The carbon paper electrode was placed in the centre of the spincoater and rotated at 5000 rpm for 60 seconds. When the spinning starts 1 mL of acetone, then 100 µL of catalytic ink solution is pipetted on the carbon paper, then it is left to rotate for the remaining time. The acetone is pipetted first to prevent clusters of ink from forming in the porous carbon paper circle. Drop casts for the RDE experiments were prepared on GC electrode without spinning.

**RDE experiments with Co dropcasts**

The electrochemical experiments involving the cobalt dropcasts were performed in a custom three-electrode cell with a electrolyte capacity of 50 mL. The WE is the aforementioned rotating glassy carbon electrode (1.96 cm^2^, Pine research) which can be rotated by a Pine instruments MSR rotator. The CE is again a flame annealed gold wire in a separate compartment with a frit between the wire and the main compartment. The reference electrode is a reversible hydrogen electrode connected with a Luggin capillary. A flame annealed platinum wire was connected to the reference electrode to act as a condenser. Before each experiment blank CV’s were made under argon and oxygen bubbling and with and without rotation at 1600 rpm.

**Convergent paired electrolysis experiments**

Convergent paired electrolysis experiments were carried out in a H-cell setup, in which the anodic and cathodic compartment were separated by an anionic exchange membrane (Forblue SELEMION AHO). An electrolyte volume of 12 mL was used in the cathodic compartment and 19 mL in the anodic compartment. The electrode used in the cathodic compartment was a flexible porous carbon paper sheet (Freudenberg H23 from FuelCellStore) with 2 cm^2^ surface area. For the anodic compartment the electrode employed was a BDD plate with 2 cm^2^ surface area. As a reference electrode either an Ag/AgCl reference electrode filled with 3 M KCl (Pine) or a reversible hydrogen reference electrode in a separate compartment connected with a Luggin capillary were used. (In case of the Ag/AgCl RE, the separate compartment was adjacent to the anode and in case of the RHE it was adjacent to the cathode; The position of the RE was found not to significantly affect the results, as control CVs of WOR at solely the BDD and the CE replaced by an Au or Pt wire in the same compartment as that of the WE in the H-cell with the RHE gave the same result as its counterpart in the H-cell with the Ag/AgCl RE). The cathodic and anodic compartments of the cell were saturated with O_2_ and Ar for 20 minutes, respectively. Prior to the introduction of the catalysts in solution, the electrode surfaces were controlled by performing 10 CVs from + 0.2 to – 0.7 V vs. Ag/AgCl for the carbon paper and from + 0.4 to + 1.7 V vs. Ag/AgCl for the BDD. For this measurement the electrode of interest was configured as a WE and an additional Au wire was inserted to be used as a CE in the CV measurement After this, **Cu(tmpa)** and **Sn-TMPyP** were added in solution and 3 CVs were performed from + 0.2 to – 0.7 V vs. Ag/AgCl at the cathode and from + 0.4 to + 1.7 V vs. Ag/AgCl at the anode. In case of the experiment with dropcast **Co-(Salphen-OH)** all modifications in the anode compartment were kept identical, while **Co-(Salphen-OH)** dropcast on carbon paper (see above) was used as a cathode instead. During the convergent paired electrolysis experiments, the BDD was set to be the working electrode while the cathode was set to be the CE. Ar and O_2_ were continuously fluxed into the anodic and cathodic compartments, respectively, during CPE experiments.

**Selection of 2e-ORR catalystst:**

A selection of cobalt catalysts was made on basis of a series of benchmarking studies carried out by the group of Stahl, wherein homogeneous cobalt catalysts were compared in terms of a log TOF versus $E_{1/2}$. Using this Bell-Evans-Polanyi comparison several cobalt catalysts were compared while operating under wildly different reaction conditions employing different solvents and proton sources. Most of the compounds studied – particularly the more suitable ones in terms of catalytic activity – are not soluble in water. We therefore opted to dropcast five representative cobalt catalysts (scheme S1) as an ink containing carbon black (3.6 mg), Nafion solution (4 μL), and the catalyst (1 μmol) in 8 mL acetone on carbon electrode. Our initial experiments illustrated that of the six Co complexes shown in Scheme S1, **Co(Salphen-OH)**, **Co(Salphen-OMe)** and **Co-TPP** performed best, especially at higher pH. We therefore opted to study dropcasts of these three aforementioned catalysts by RDE experiments under relevant conditions (0.1 M carbonate buffer, pH 9.2), allowing for direct comparison of onset potential, overpotential, Faradaic efficiencies and Tafel slopes. Therefore **Co-OEP**, **Co(Salen)** and **Co-TMPyP** were discarded in further experiments.

We studied drop casts of **Co(Salphen-OH)**, **Co(Salphen-OMe)** and **Co-TPP** by RDE experiments under relevant reaction conditions (0.1 M carbonate buffer at pH 9.2), allowing for direct comparison of onset potential, overpotential, Faradaic efficiencies and Tafel slopes.

**Scheme S1**. Structures of Co-complexes considered in this mansucript.

**Table S2.** Catalytic data for **Co(Salphen-OH)**, **Co(Salphen-OMe)** and **Co-TPP** in 2e-ORR RDE experiments.

|  | Tafel slope^[a]^ (mV/dec) | Tafel slope after CPE^[b]^ (mV/dec) | Onset Potential^[c]^ (V vs RHE) |
| --- | --- | --- | --- |
| **Co-TPP** | 90 | 117 | 0.68 |
| **Co(Salphen-OMe)** | 148 | 104 | 0.79 |
| **Co(Salphen-OH)** | 66 | 67 | 0.82 |

^[a]^ Tafel slope recorded by LSV (1600 rpm , 1.5 mV/s); ^[b]^ Tafel slope recorded by LSV (1600 rpm , 1.5 mV/s) after one hour of CPE (1 mA/cm^2^); ^[c]^ Onset potential is defined as the potential which the current density exceeds 10 μA/cm^2^ after a background correct.

**Co(Salphen-OH)** was selected as the most suitable catalyst to be employed in controlled paired electrolysis experiments given that it shows excellent catalytic activity with a low overpotential, while the catalytic activity is maintained after one hour of constant potential electrolysis. In a controlled paired electrolysis configuration wherein **Co(Salphen-OH)** was dropcast on carbon paper in the cathode compartment and **Sn-TMPyP** was dissolved in the anode compartment employing a BDD WE a H_2_O_2_ yield of 0.58 µmol h^-1^ cm^-2^ ($\mathrm{FE}_{H_{2}O_{2}}$ 29%) at the anode, 1.07 µmol h^-1^ cm^-2^ ($\mathrm{FE}_{H_{2}O_{2}}$ 50%) at the cathode, and an overall yield of 1.65 µmol h^-1^ cm^-2^ was obtained during a 3 hour experiment. See Figures S13 – S15.

**KIE experiments**

Prior to all kinetic isotopic experiments, all glassware was placed in an oven at 120 °C overnight. A 100 mM phosphate buffer solution was prepared in D_2_O, and the pH was measured using a H_2_O-calibrated pH meter. The measured pH of the D_2_O solution was converted according to the equation:

$pH=0.929\times\mathrm{pH}_{D_{2}O}+0.4$1

obtaining a $\mathrm{pH}_{D_{2}O}$ of 9.2. The electrodes were prepared as described above, and all the electrodes were rinsed with D_2_O prior to the measurement. The surface of the WE was saturated by the correct D_2_O fraction by measuring 10 CV cycles in a blank solution in absence of catalyst prior to recording the catalytic activity.

**KMnO_4_ titration for the quantification of H_2_O_2_**

The H_2_O_2_ produced during CPE and paired electrolysis experiments employing **Sn-TMPyP** was quantified through permanganate titration according to the reaction:

2 KMnO_4_  + 5 H_2_O_2_ + 3 H_2_SO_4_ → 2 MnSO_4_  + 5 O_2_ + K_2_SO_4_ + 8 H_2_O

The titration reaction was followed by means of UV-Vis spectroscopy, following the decrease of absorbance of KMnO_4_ at 525 nm (ε = 2450 M^-1^ cm^-1^).^[10]^ The amount of H_2_O_2_ was quantified according to the stoichiometry of the reaction. During a typical experiment, 1.5 mL of electrolyte was extracted from the electrochemical cell and filtered over porous silica (60-200 Å pore dimension) to remove any residue of **Sn-TMPyP** and avoid any interference leading to the overestimation of the H_2_O_2_ content of the solution. The filtration step was only performed with **Sn-TMPyP** in the reaction mixture and not for the catholyte solutions. 1 mL of the filtered solution was inserted into a 1 cm path length cuvette and diluted with 1.95 mL of MilliQ. 100 μL of 1 M H_2_SO_4_ were added and the blank spectrum was recorded. Then 10 μL of a 15 mM stock solution of KMnO_4_ were added in the cuvette under stirring (leading to a formal KMnO_4_ concentration of 50 μM) and the spectrum was recorded after 60s. For the quantification of higher concentrations of H_2_O_2_ the procedure was repeated adding more KMnO_4_ until completion of the titration. Prior to the quantification of the produced H_2_O_2_, a blank test was performed in absence of H_2_O_2_ to analytically assess the concentration of KMnO_4_ introduced within each injection.

**Ce(SO_4_)_2_ titration for the quantification of H_2_O_2_**

Due to presence of carbonate buffer, the addition of insufficient H_2_SO_4_ may lead to formation of deposits during the permanganate titration which may interfere with the quantification of H_2_O_2_. Particularly for the RDE experiments with Co-catalysts, a cerimetry procedure^[11]^ was used to quantify the amount of H_2_O_2_ formed.

As cerium source, cerium sulfate was chosen which reacts with peroxide according to the following reaction:

$$2 Ce{(SO_{4})}_{2}+ H_{2}O_{2} \to\mathrm{Ce}_{2}{{(SO}_{4})}_{3}+ H_{2}\mathrm{SO}_{4}+ O_{2}$$

The titration was followed by UV-vis, wherein the decrease of absorbance by Ce(SO_4_)_2_ at 255 nm was monitored. From the decrease in absorbance the concentration of H_2_O_2_ can be determined from a calibration curve generated from a series of experiments with samples of known H_2_O_2_ concentration and the same cerium sulfate concentration. In a typical experiment, a sample of 1 mL was taken from the cell and 1.95 mL of MilliQ was added together with a 50 µL of 1M sulfuric acid in MilliQ. This solution was then transferred to a cuvette with a pathlength of 1 cm and a blank spectrum was recorded. Afterwards 10 µL of a 15 mM solution of cerium sulfate in MilliQ was added and the solution was sonicated for one minute, after which another UV-vis spectrum was recorded.


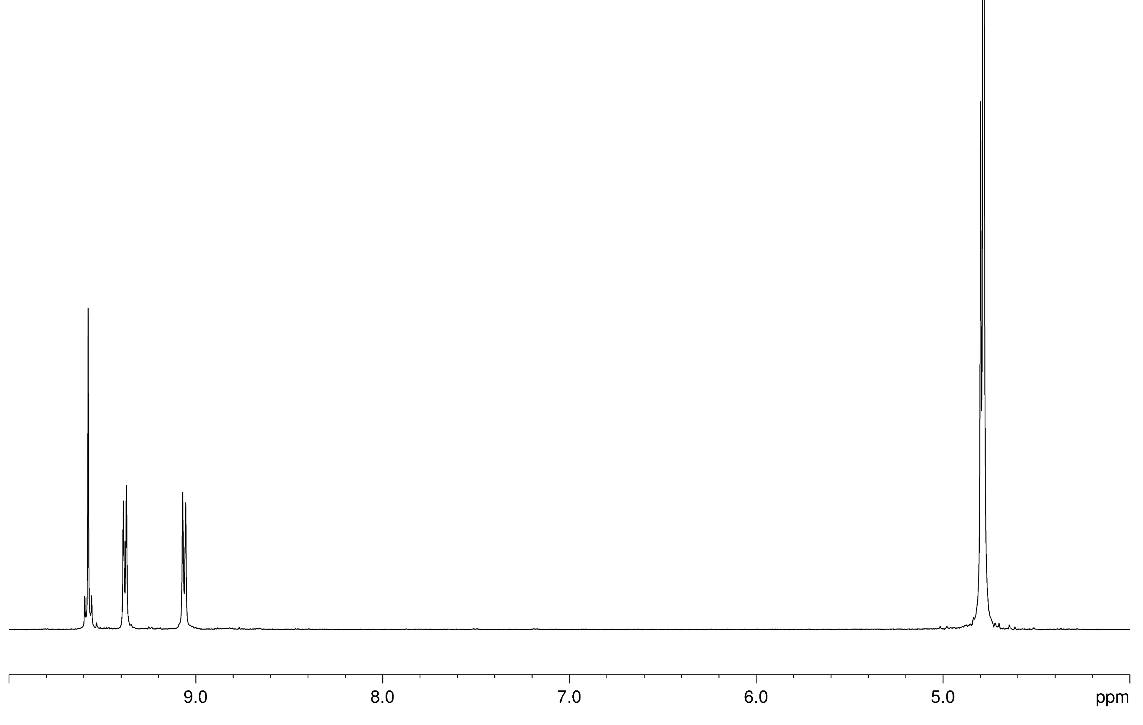


**Figure S1.** ^1^H NMR spectrum of **Sn-TMPyP** (D_2_O, 298 K)


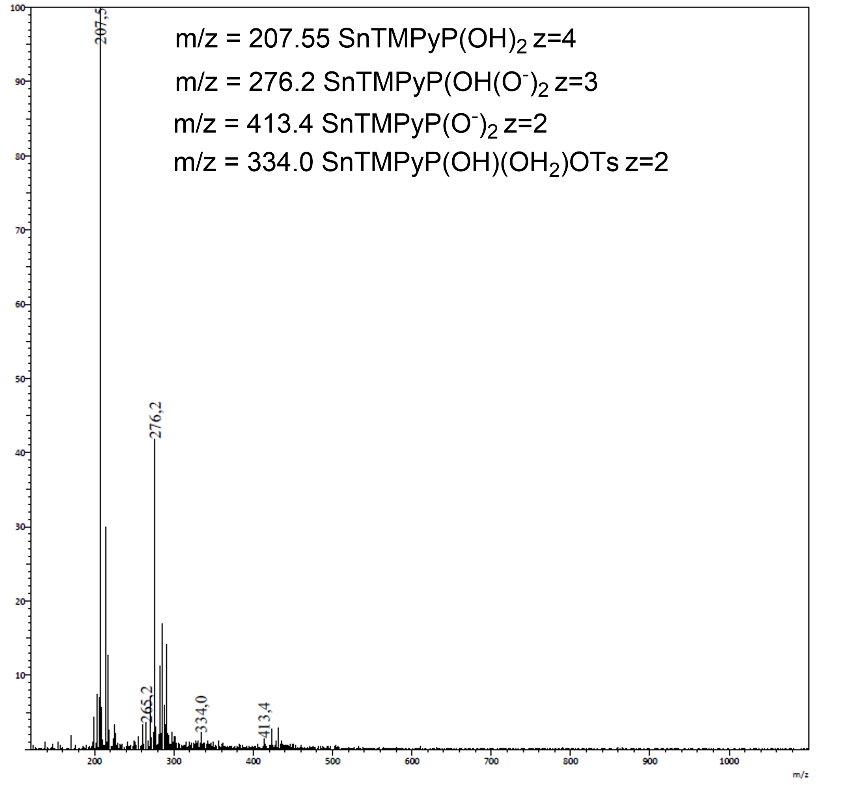


**Figure S2.** LC-MS spectrum of **Sn-TMPyP** in D_2_O and species associated to the main peaks.

**Table S1.** Crystallographic data for **Sn-TMPyP.**

|  | **Sn-TMPyP** |
| --- | --- |
| Crystal data | |
| Chemical formula | C_44_H_36_Cl_2_N_8_Sn·5.28(CH_4_O)·4(Cl) |
| *M*_r_ | 1177.38 |
| Crystal system, space group | Monoclinic, *P*2_1_/*c* |
| Temperature (K) | 223 |
| *a*, *b*, *c* (Å) | 8.85853 (4), 25.67478 (11), 14.41056 (6) |
| β (°) | 93.9826 (4) |
| *V* (Å^3^) | 3269.63 (2) |
| *Z* | 2 |
| Radiation type | Cu *K*α |
| μ (mm^-1^) | 5.72 |
| Crystal size (mm) | 0.22 × 0.13 × 0.10 |
|  | |
| Data collection | |
| Diffractometer | XtaLAB Synergy R, HyPix |
| Absorption correction | Analytical  *CrysAlis PRO* 1.171.42.95a (Rigaku Oxford Diffraction, 2023) Analytical numeric absorption correction using a multifaceted crystal model based on expressions derived by R.C. Clark & J.S. Reid.^[12]^ Empirical absorption correction using spherical harmonics, implemented in SCALE3 ABSPACK scaling algorithm. |
| *T*_min_, *T*_max_ | 0.426, 0.646 |
| No. of measured, independent and  observed [*I* > 2σ(*I*)] reflections | 65483, 6412, 6056 |
| *R*_int_ | 0.019 |
| (sin θ/λ)_max_ (Å^-1^) | 0.617 |
|  | |
| Refinement | |
| *R*[*F*^2^ > 2σ(*F*^2^)], *wR*(*F*^2^), *S* | 0.033, 0.102, 1.07 |
| No. of reflections | 6412 |
| No. of parameters | 331 |
| H-atom treatment | H-atom parameters constrained |
| Δρ_max_, Δρ_min_ (e Å^-3^) | 0.97, -0.56 |


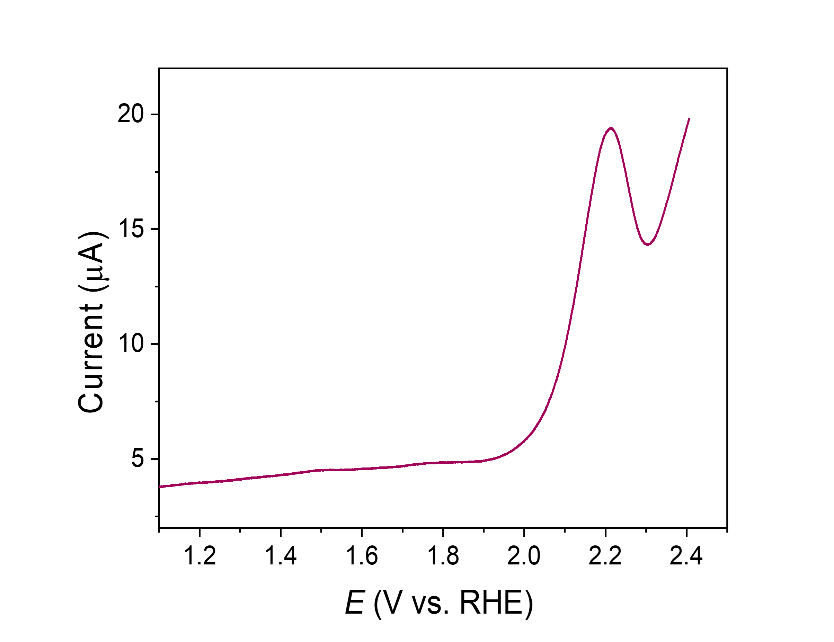


**Figure S3.** DPV of **Sn-TMPyP** (0.2 mM) in 0.1 M Na_2_SO_4_ + 7.4 mM Na_2_HPO_4_ at pH 8.5 under Ar. GC WE, Au CE, Ag/AgCl RE.

**
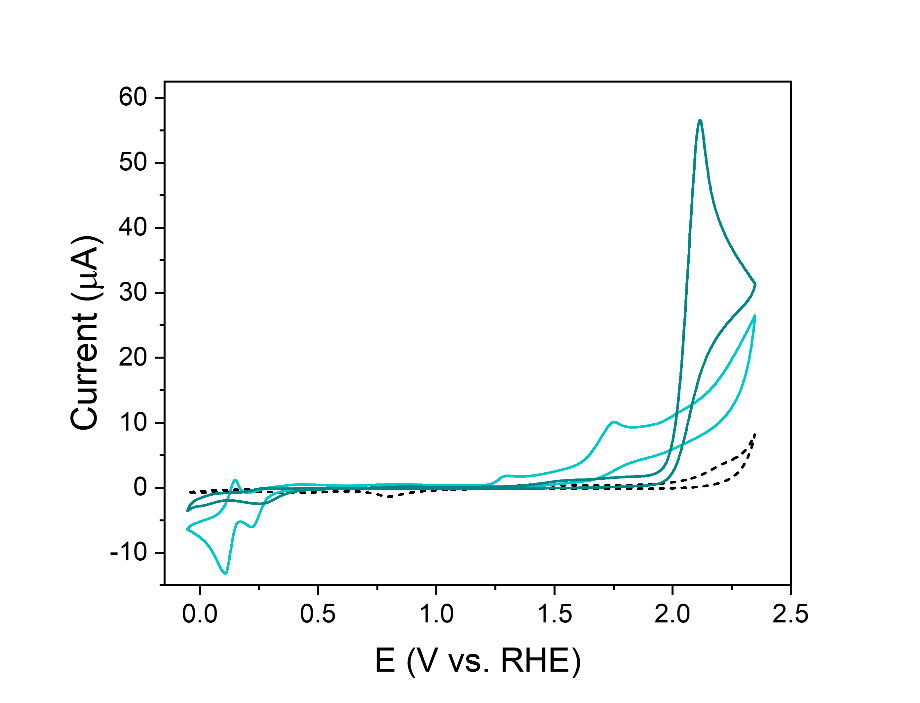
**

**Figure S4.** CV of 0.2 mM **Sn-TMPyP** (green) and H_2_TMPyP tosylate (dark green) at 100 mV s^−1^ scan rate recorded in 0.1 M carbonate buffer pH at 9.2 under Ar.


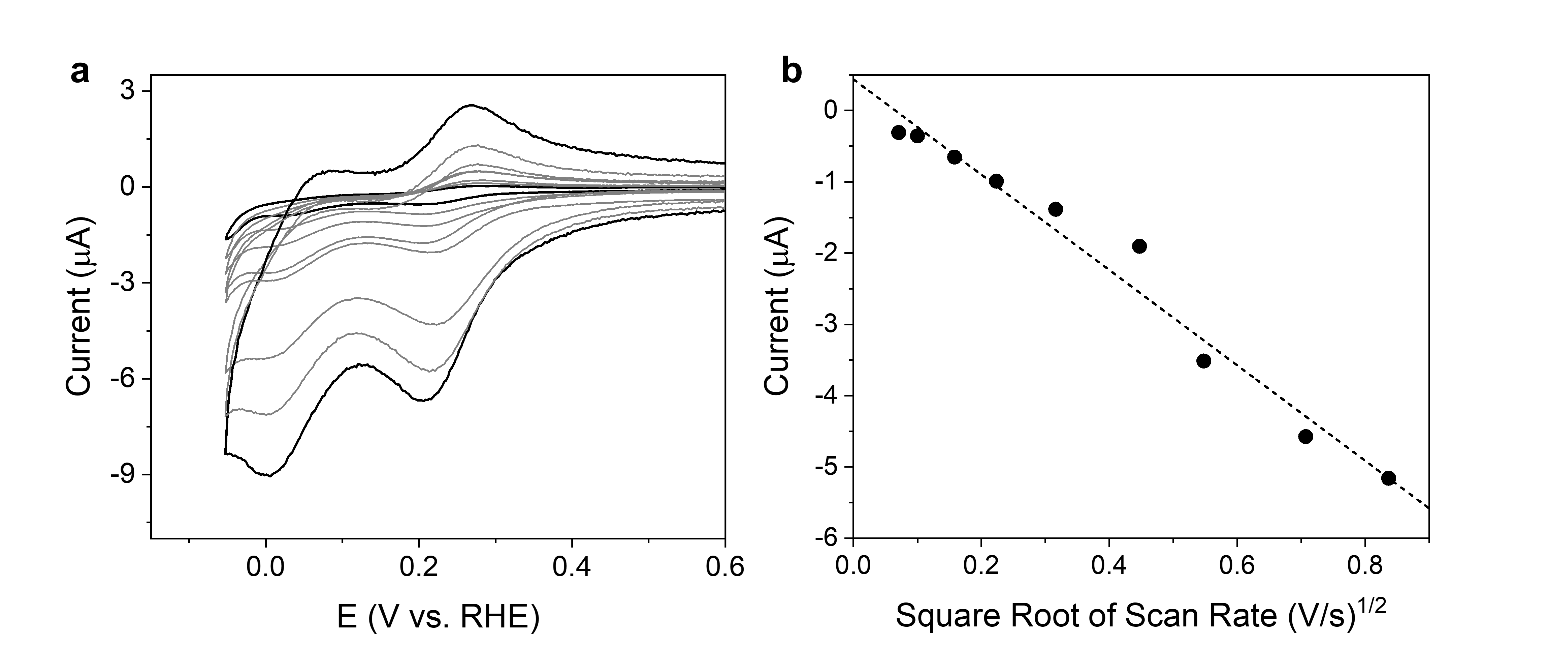


**Figure S5.** a) CVs of 0.2 mM **Sn-TMPyP** at various scan rates recorded in 0.1 M carbonate buffer at pH 9.2 under Ar. b) Plot of the cathodic peak currents as a function of the square root of the scan rate.


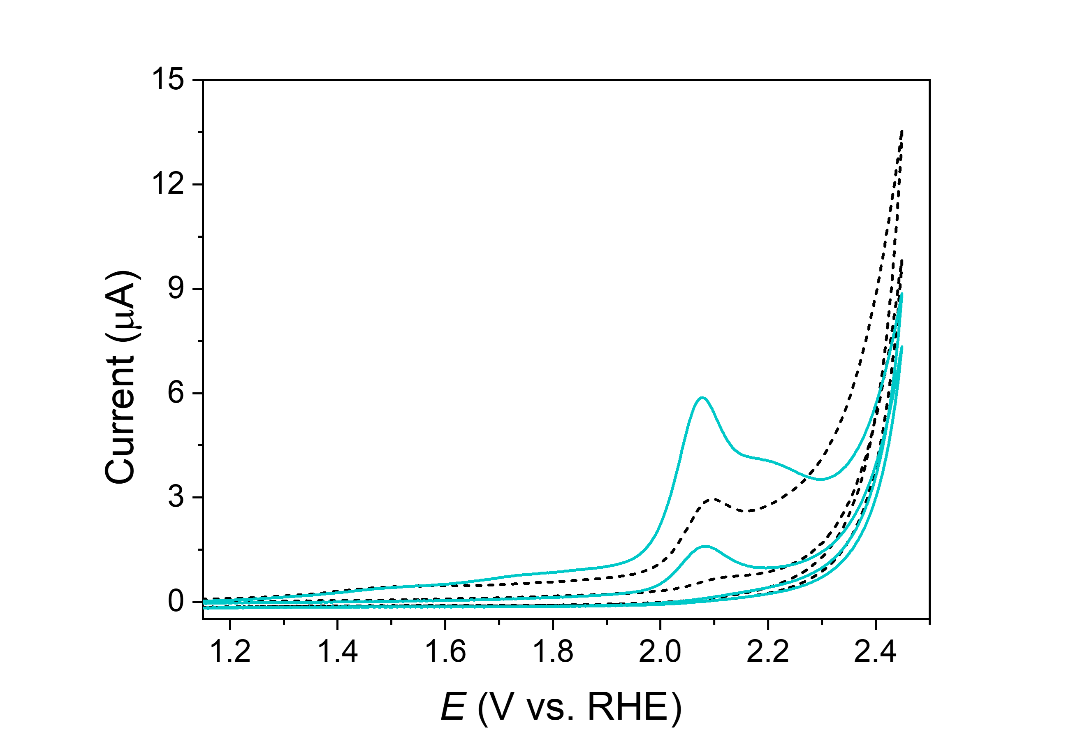


**Figure S6.** CVs of a rinse test performed after the electrode was immersed in a solution 0.2 mM **of Sn-TMPyP** and after recording 25 CVs in it. Thereafter the BDD electrode was thoroughly rinsed with Mili-Q water and used to record CVs in a solution of 0.1 M carbonate buffer at pH 9.2 under Ar (blue) and compared to the blank CVs (black).


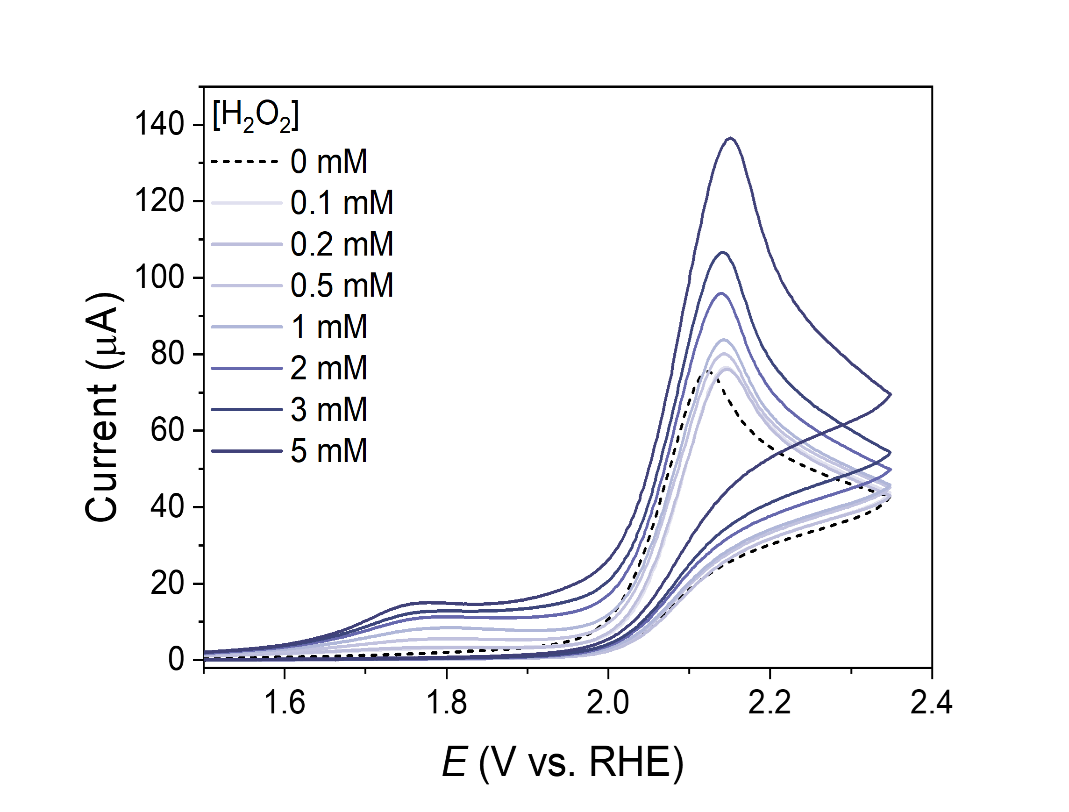


**Figure S7.** CVs of 0.2 mM **Sn-TMPyP** at 100 mVs^-1^ in presence of increasing concentration of H_2_O_2_ in 0.1 M carbonate buffer at pH 9.2 under Ar.


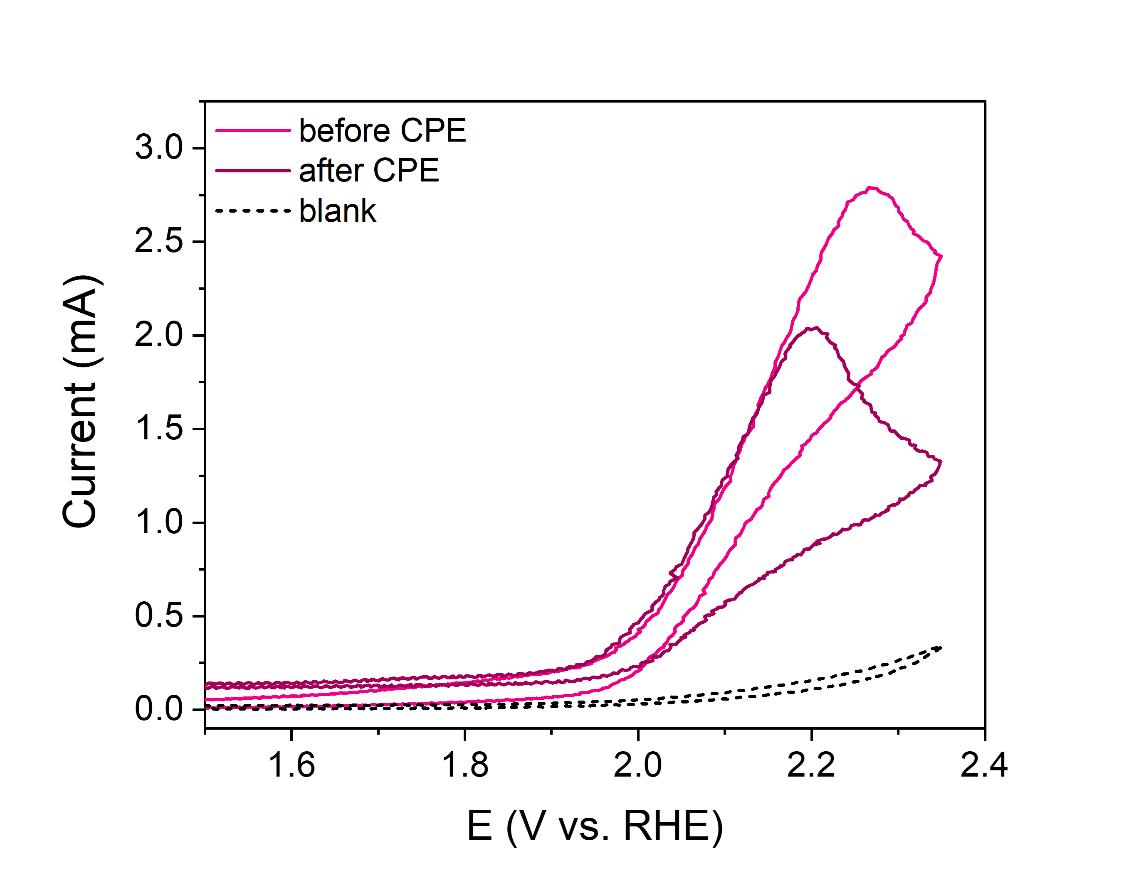


**Figure S8.** CVs of 0.2 mM **Sn-TMPyP** recorded before and after 4 h CPE at 2.1 V vs. RHE on a 2 cm^2^ BDD electrode in 0.1 M carbonate buffer pH at 9.2 at 100 mVs^-1^ under Ar.


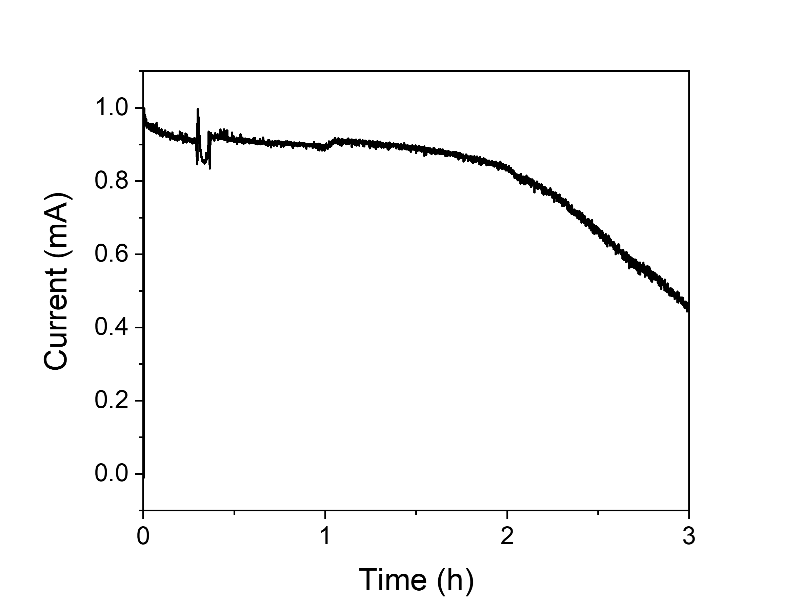


**Figure S9.** Amperogram of a 3h CPE at 2.15 V vs. RHE with 0.2 mM **Sn-TMPyP** at the anode under Ar and 0.2 mM **Cu(tmpa)** at the cathode under O_2_ in 0.1 M carbonate buffer at pH 9.2.


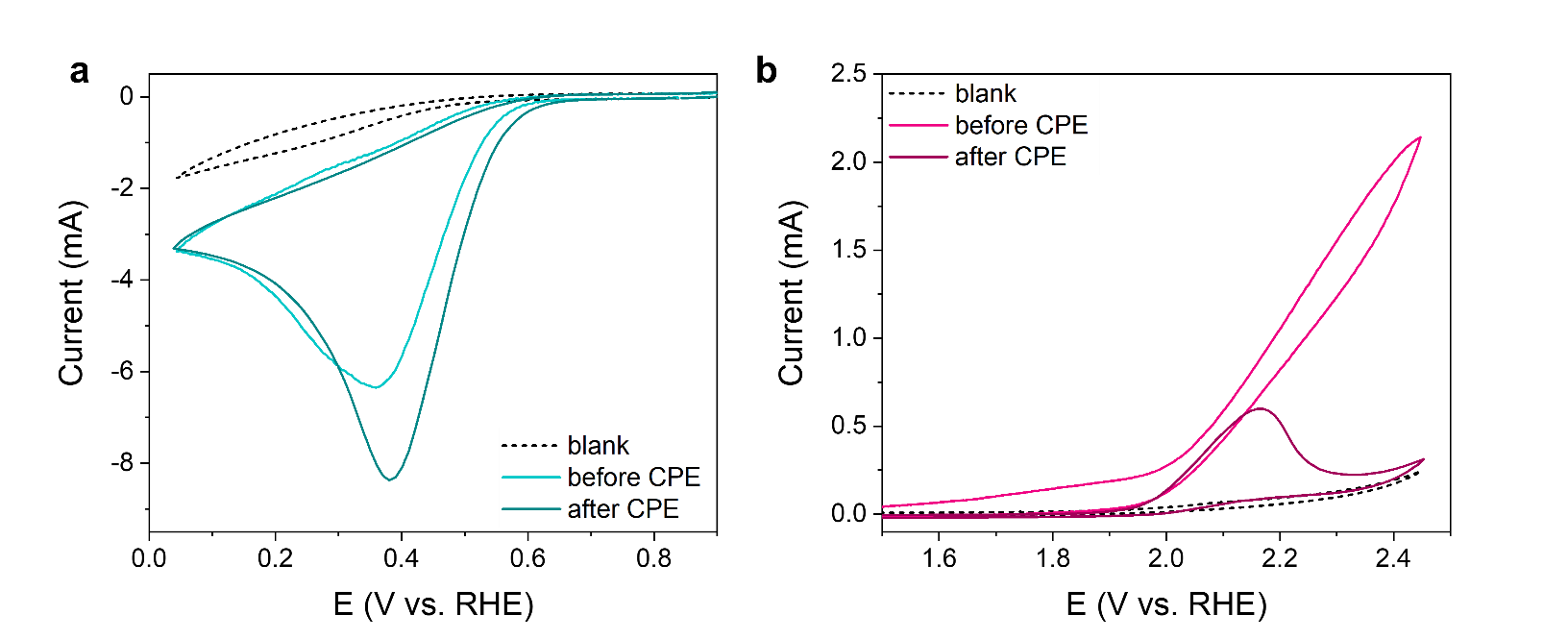


**Figure S10.** a) CVs of 0.2 mM **Cu(tmpa)** recorded before and after CPE on a 2 cm^2^ carbon paper electrode in 0.1 M carbonate buffer at pH 9.2 at 100 mVs^-1^ under O_2_. b) CVs of 0.2 mM **Sn-TMPyP** recorded before and after CPE at 2.15 V vs. RHE on a 2 cm^2^ BDD electrode in 0.1 M carbonate buffer at pH 9.2 at 100 mVs^-1^ under Ar.


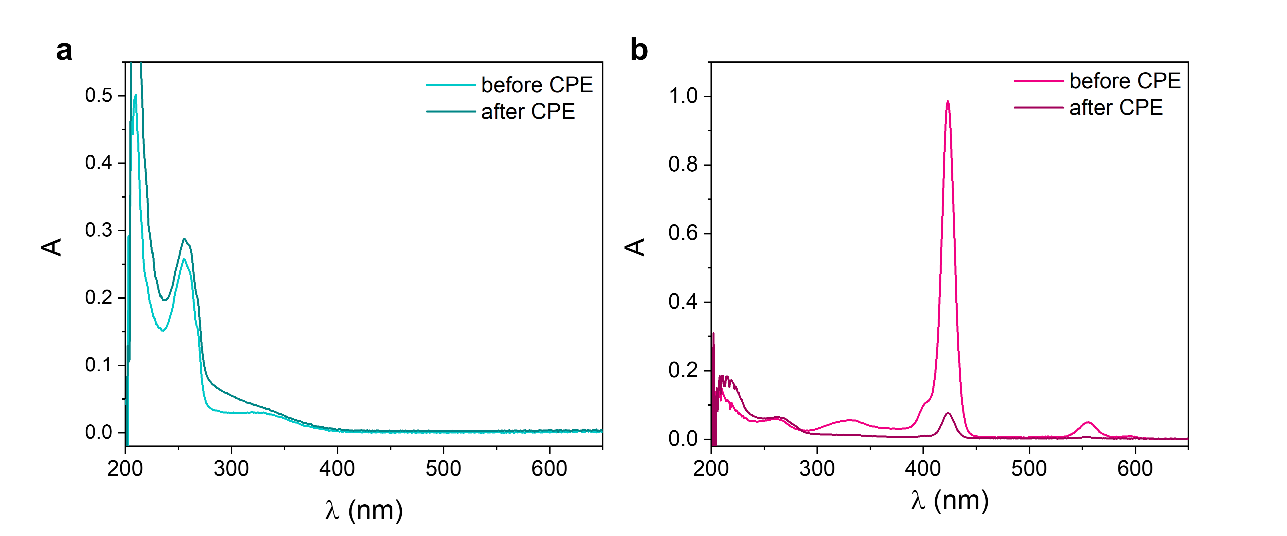


**Figure S11.** a) UV-Vis spectra of 0.02 mM **Cu(tmpa)** recorded before and after on a 2 cm^2^ carbon paper electrode in 0.1 M carbonate buffer at pH 9.2 under O_2_. b) UV-Vis spectra of 4 μM **Sn-TMPyP** recorded before and after CPE at 2.15 V vs. RHE on a 2 cm^2^ BDD electrode in 0.1 M carbonate buffer at pH 9.2 under Ar.


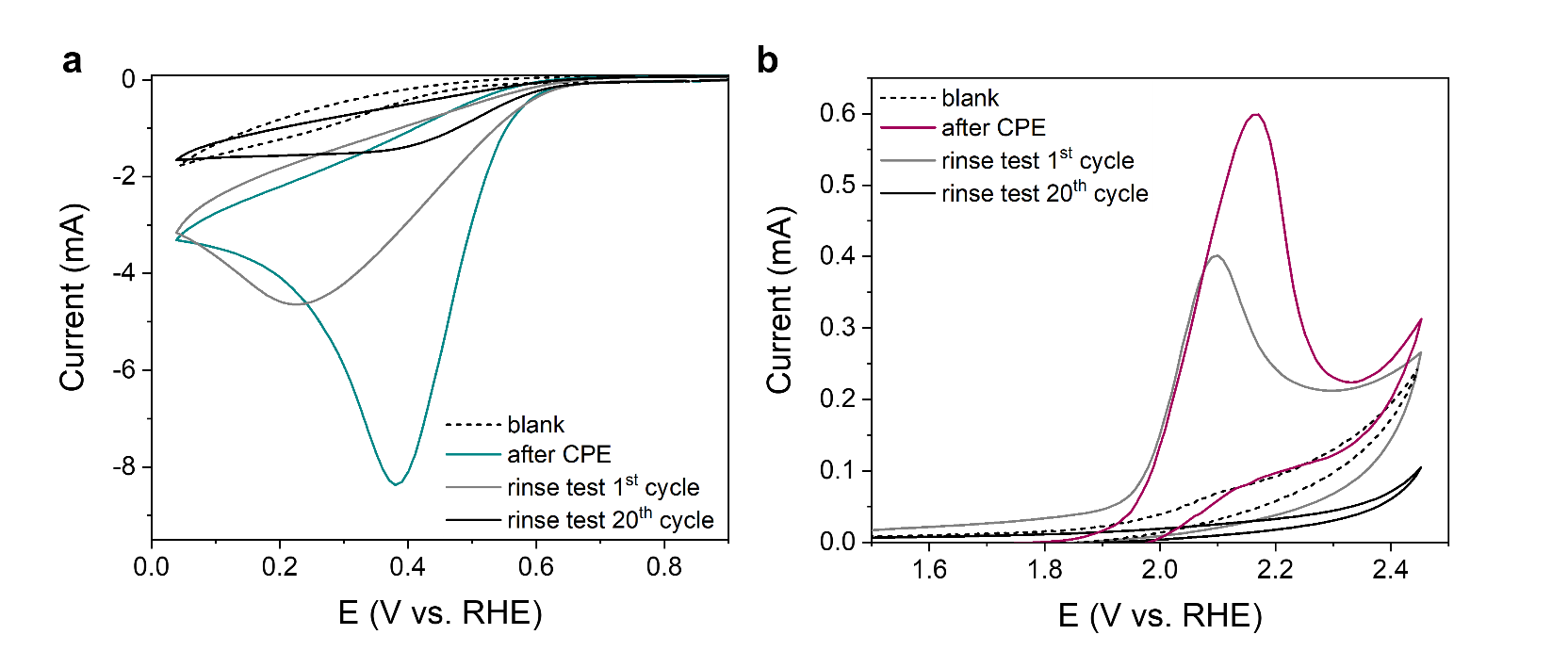


**Figure S12.** a) CVs of 0.2 mM **Cu(tmpa)** recorded after CPE at 2.15 V vs. RHE on a 2 cm^2^ carbon paper electrode in 0.1 M carbonate buffer at pH 9.2 under O_2_ and rinse tests. b) CVs of 0.2 mM **Sn-TMPyP** recorded after CPE at 2.15 V vs. RHE on a 2 cm^2^ BDD electrode in 0.1 M carbonate buffer at pH 9.2 under Ar and rinse tests.


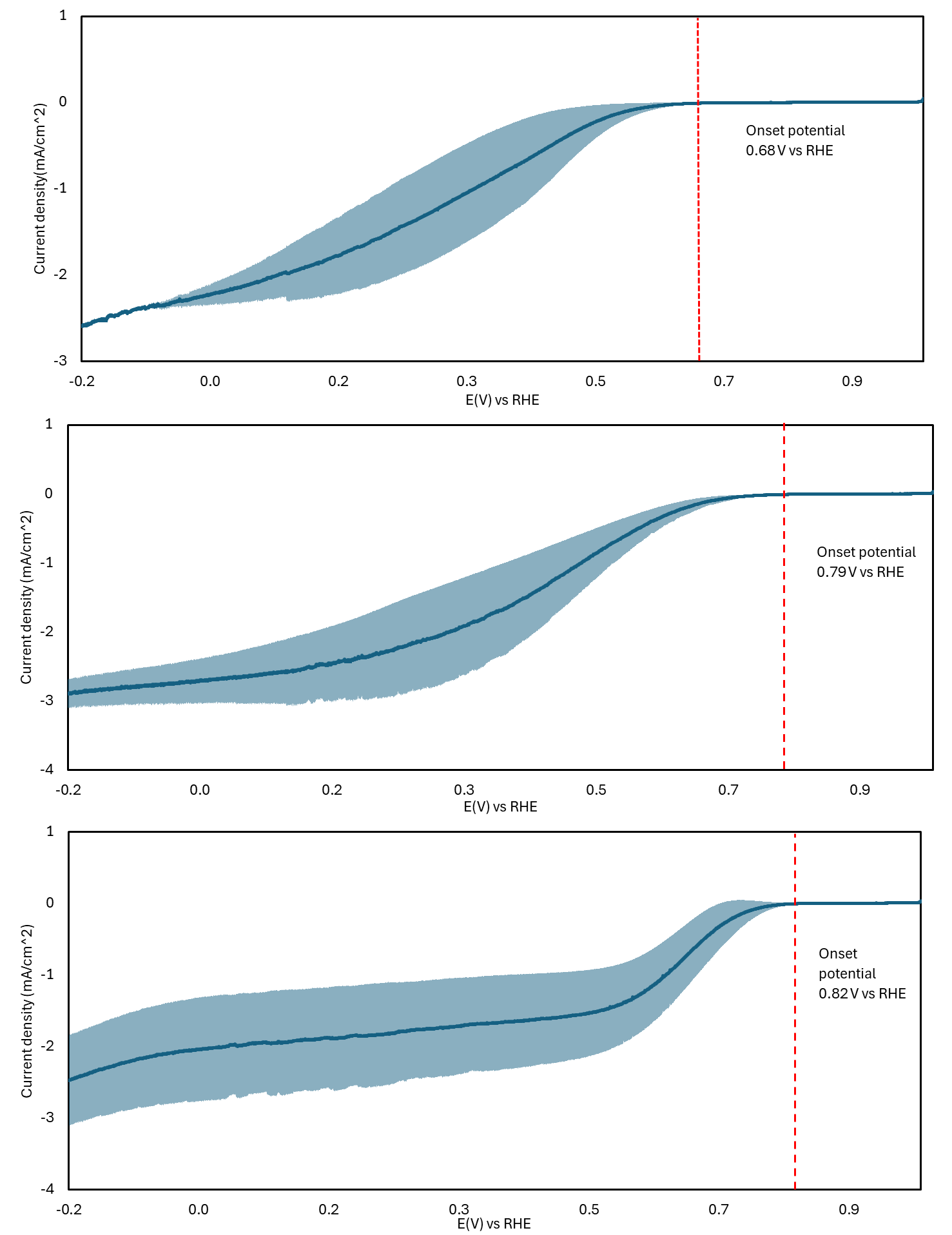


**Figure S13** Onset potentials of **Co-TPP**, **Co(Salphen-OMe)** and **Co(Salphen-OH)** inks with shaded area showing twice the sample standard deviation of the three measurements. All measurements were done in triplicate (n=3) with the average forming the solid line and were performed under oxygen bubbling using linear sweep voltammetry with a rotating glassy carbon electrode in a carbonate buffer (0.1 M pH 9.2) at 1600 rpm with a scan speed of 1.5 mV/s and a step of 0.15 mV .


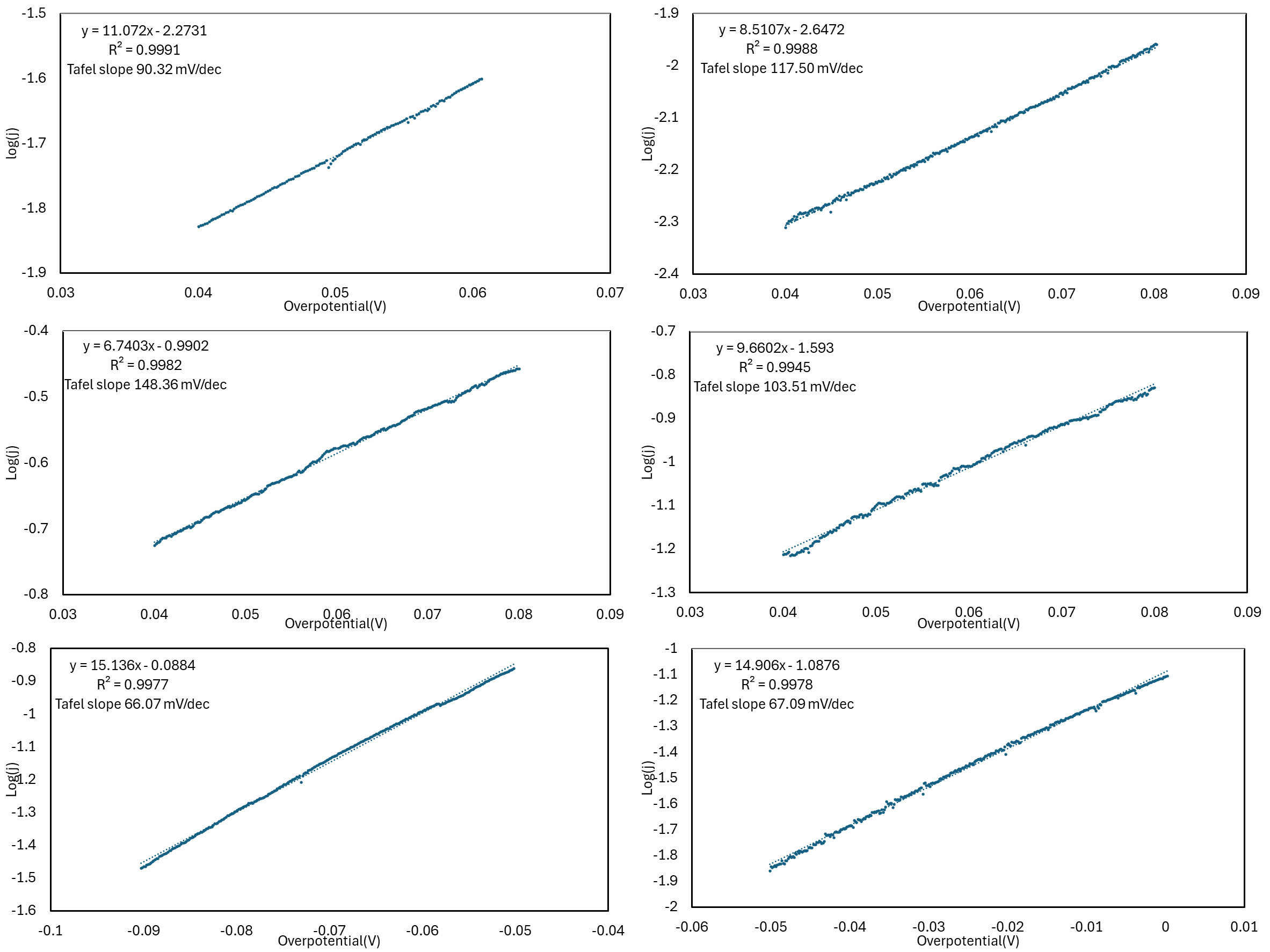


**
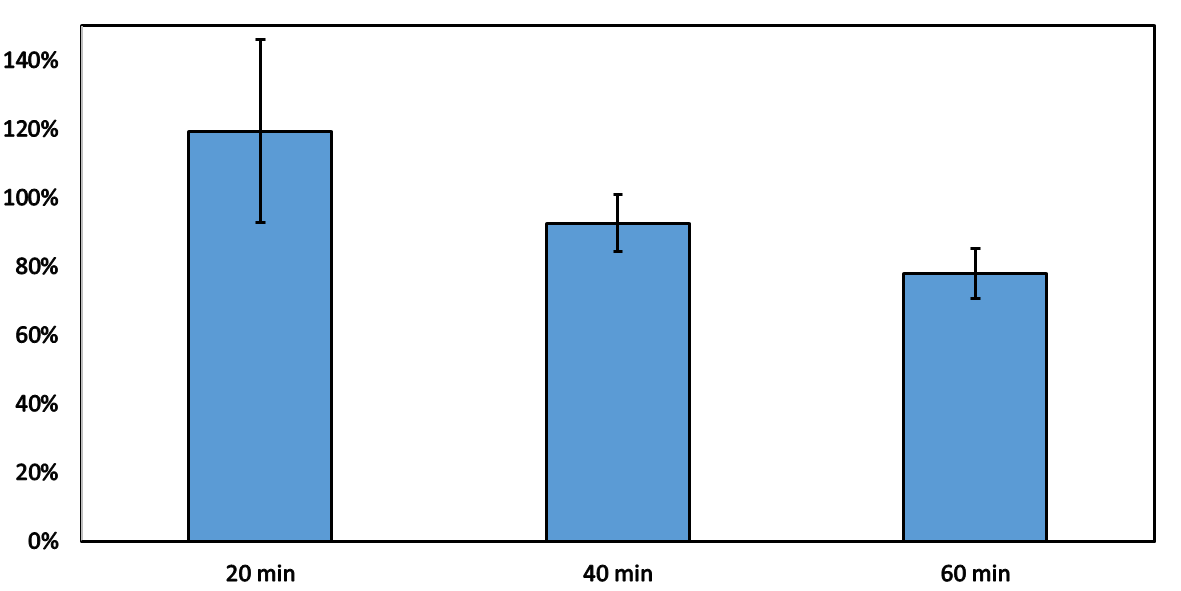

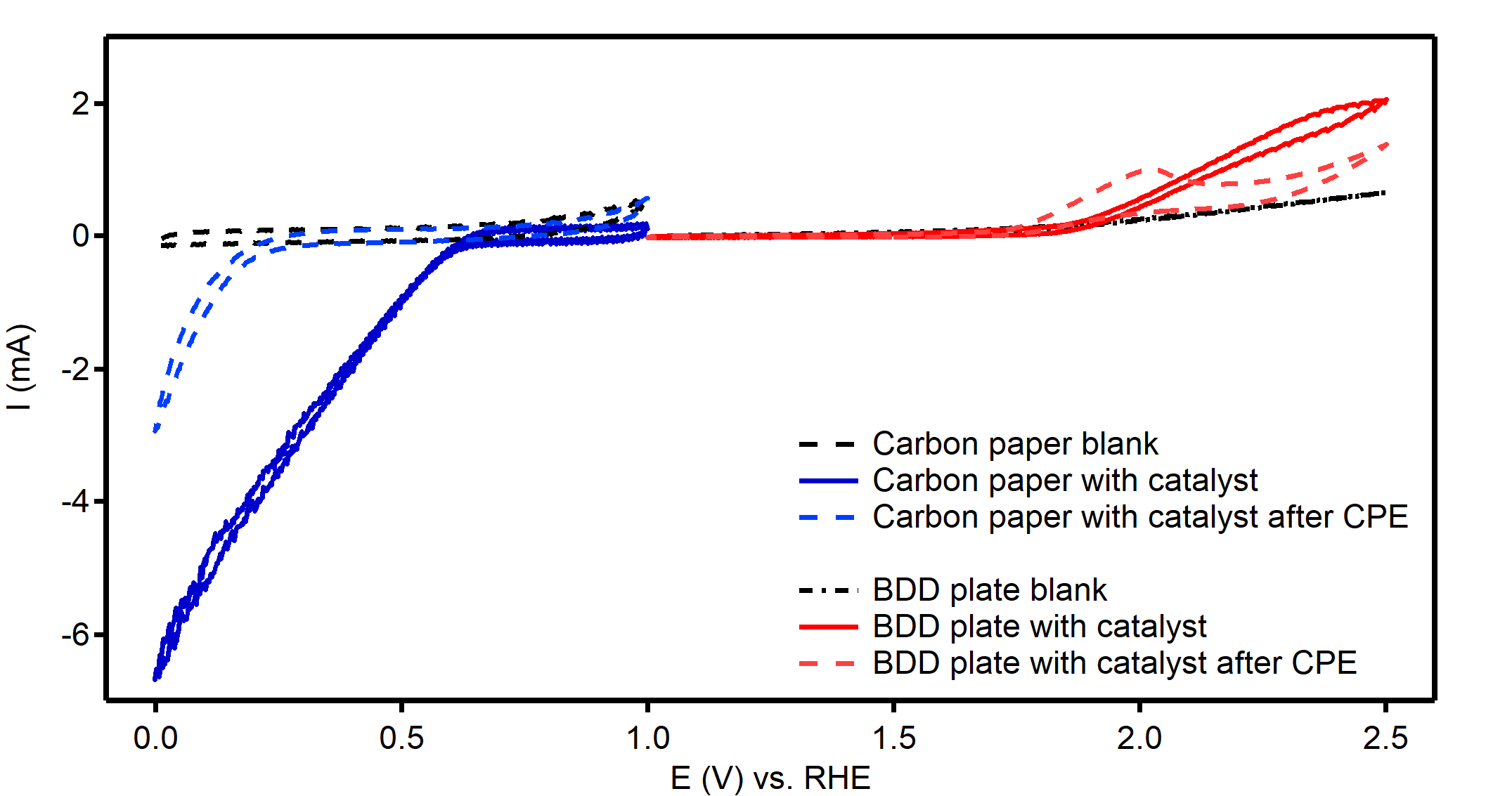
**

**Figure S14** left, the Tafel slopes of the different cobalt catalyst inks (**Co-TPP**, **Co(Salphen-OMe)** and **Co(Salphen-OH)**) before 1 h of chronoamperometry at 1 mA/cm^2^. Right, the Tafel slopes of the different cobalt catalysts (**Co-TPP**, **Co(Salphen-OMe)** and **Co(Salphen-OH)**) after 1 h of chronoamperometry at 1 mA/cm^2^. All measurements were done in triplicate (n=3) with the average being shown and were performed under oxygen bubbling using linear sweep voltammetry with a rotating glassy carbon electrode in carbonate buffer (0.1 M pH 9.2) at 1600 rpm with a scan speed of 1.5 mV/s and a step of 0.15 mV

**Figure S15** Faradaic efficiency, with error bars showing the standard deviation, of hydrogen peroxide production by the two-electron ORR of **Co(Salphen-OH)** inks in intervals of 20 minutes. Measurements at 20 min performed in duplicate (n=2) at 40 and 60 min performed in triplicate (n=3) with the average being shown and were obtained during CA with a current of 1 mA/cm^2^ with a rotating glassy carbon electrode in 0.1 M carbonate buffer at pH 9.2 at 1600 rpm.

**Figure S15** In blue, CV scans of the spin coated **Co(Salphen-OH)** on carbon paper before and after controlled paired electrolysis and a blank without spin coated catalyst. All in 0.1 M carbonate buffer at pH 9.2 during oxygen bubbling and stirring at 1000 rpm. In red, CV scans of the BDD plate with and without 0.2 mM **Sn-TMPyP** catalyst in the electrolyte solution and a blank without catalyst in solution. All in 0.1 M carbonate buffer at pH 9.2 during argon bubbling and stirring at 1000 rpm.

**
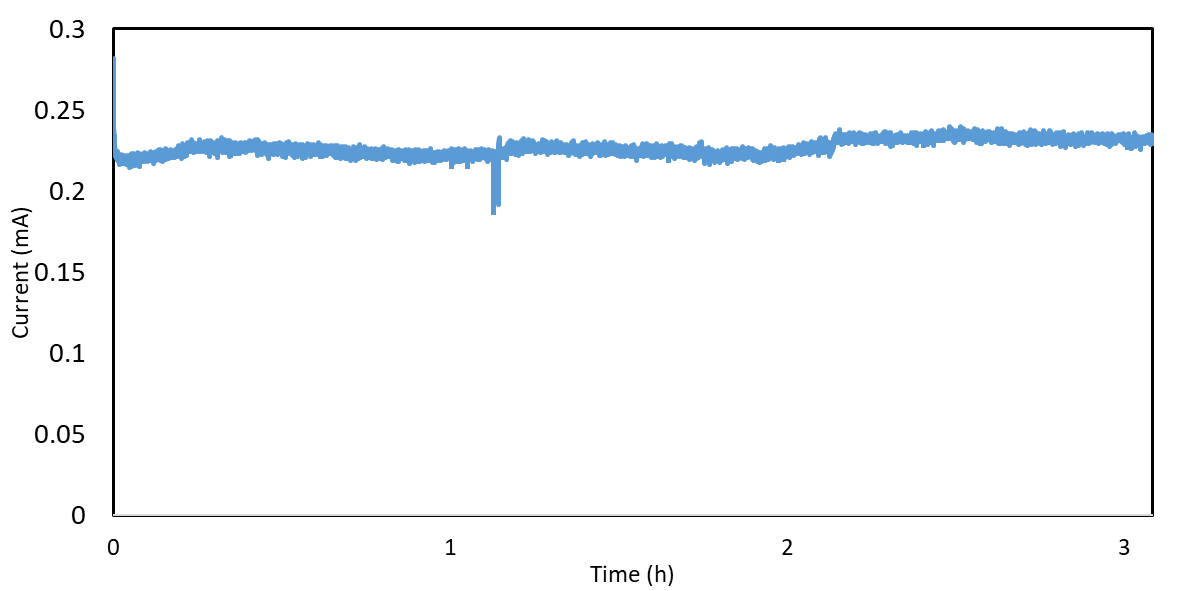
**

**Figure S16** Amperogram of controlled paired electrolysis over 3 h at 2.2 V vs RHE set at the anode. Anode: a 2 cm^2^ BDD electrode in presence of 0.2 mM **Sn-TMPyP** in carbonate buffer (0.1 M pH 9.2), argon bubbling 1000 rpm stirring. Cathode: a 2 cm^2^ carbon paper electrode coated with 100 μL **Co(Salphen-OH)** ink solution in 0.1 M carbonate buffer at pH 9.2, oxygen bubbling 1000 rpm stirring.

**
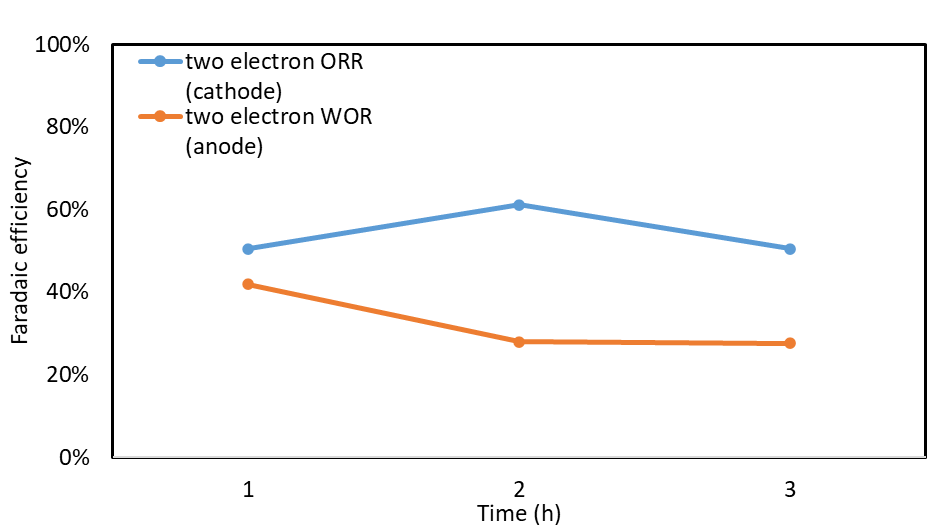

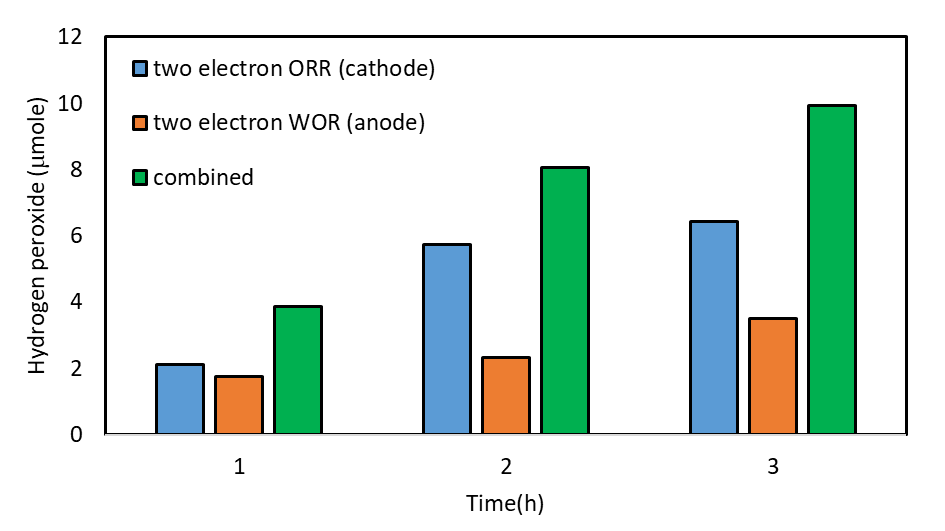
**

**Figure S18** Faradaic efficiency of the controlled paired electrolysis experiment of the cathode (blue) and the anode (orange). Anode: a 2 cm^2^ BDD electrode in 0.2 mM Sn-TMPyP in 0.1 M carbonate buffer at pH 9.2, argon bubbling, 1000 rpm stirring. Cathode: a 2 cm^2^ carbon paper electrode coated with 100 μL **Co(Salphen-OH)** ink solution in 0.1 M carbonate buffer at pH 9.2, 2.2 V vs RHE set at the anode for 3 h. oxygen bubbling, 1000 rpm stirring.

**Figure S17** The amount of hydrogen produced at the cathode, anode and the whole cell combined during a 3 h controlled paired electrolysis experiment. Anode: a 2 cm^2^ BDD electrode in 0.2 mM **Sn-TMPyP** in 0.1 M carbonate buffer at pH 9.2 ,argon bubbling 1000 rpm stirring. Cathode: a 2 cm^2^ carbon paper electrode coated with 100 μL **Co(Salphen-OH)** ink solution in 0.1 M carbonate buffer at pH 9.2, 2.2 V vs RHE set at the anode for 3 h, oxygen bubbling 1000 rpm stirring.

**References**

[1] M. Langerman, D. G. H. Hetterscheid, *Angew. Chem. Int. Ed.* **2019**, *58*, 12974.

[2] A. Thomas, F. Kuttassery, S. N. Remello, S. Mathew, D. Yamamoto, S. Onuki, Y. Nabetani, H. Tachibana, H. Inoue, *Bull. Chem. Soc. Jpn.* **2016**, *89*, 902.

[3] Y. H. Wang, M. L. Pegis, J. M. Mayer, S. S. Stahl *J. Am. Chem. Soc.* **2017**, *139*, 16458-16461.

[4] M. F. Lin, L. G. Marzili, *Inorg. Chem.* **1994**, *33*, 5309-5315.

[5] I. Usón, G. M. Sheldrick, *Acta Cryst D* **2018**, *74*, 106.

[6] A. L. Spek, *Acta Cryst D* **2009**, *65*, 148.

[7] J. V. Macpherson, *Phys. Chem. Chem. Phys.* **2015**, *17*, 2935.

[8] R. F. Brocenschi, K. Irikura, N. Wachter, G. M. Swain, R. C. Rocha-Filho, *Diamond Relat. Mater*. **2023**, *136*, 110008.

[9] I. Duo, C. Levy-Clement, A. Fujishima, C. Comninellis, *J. F. Appl. Electrochem.* **2004**, *34*, 935.

[10] N. V. Klassen, David. Marchington, H. C. E. McGowan, *Anal. Chem.* **1994**, *66*, 2921.

[11] F. Sandri, M. Danieli, M. Zecca, P. Centomo, *Chemcatchem* **2021**, *13*, 2653-2663.

[12] R. C. Clark, J. S. Reid, *Acta Cryst*.**1995**, *A51*, 887.
